# Isolating and Determining the Structures of Colored Products from the Reactions of Cannabinoids with Fast Blue RR

**DOI:** 10.3390/molecules30173462

**Published:** 2025-08-22

**Authors:** Kayo Nakamura, Hikari Nishiguchi, Ryosuke Arai, Riho Hamajima, Hiroko Abe, Akihiko Ishida, Manabu Tokeshi, Kyohei Higashi, Akiyoshi Saitoh, Hideyo Takahashi

**Affiliations:** 1 Faculty of Pharmaceutical Sciences, Tokyo University of Science, 6-3-1 Niijuku, Katsushika-ku, Tokyo 125-8585, Japan; 2 BioDesign Inc., IB Daiichi-Bld 6th Floor, 3-25-15 Nishi-Ikebukuro, Toshima-ku, Tokyo 171-0021, Japan; 3 Faculty of Engineering, Hokkaido University, Kita 13, Nishi 8, Kita-ku, Sapporo 060-8628, Japan

**Keywords:** cannabis, color reaction, diazo coupling, Fast Blue RR, cannabinoid, cannabidiol, cannabinol, Δ^9^-tetrahydrocannabinol

## Abstract

Although cannabis is used in a wide range of fields, including medicine and pharmacology, its use is prohibited in Japan because it contains Δ^9^-tetrahydrocannabinol (Δ^9^-THC), a compound that exhibits narcotic effects. While cannabis is primarily detected via color-based screening methods at crime scenes, the reaction products and mechanisms associated with these screening methods have not been fully elucidated. To address this issue, the colored products were isolated via the diazo-coupling reactions of the major cannabinoids (cannabidiol, cannabinol, and Δ^9^-THC) in cannabis with the Fast Blue RR diazonium salt, and their structures were determined using NMR spectroscopy. As expected, azo compound **2** was formed from cannabidiol, whereas cannabinol and Δ^9^-THC produced quinoneimines **3** and **4**, respectively. This study is expected to lead to the future development of more sensitive color-based reagents that produce fewer false positives.

## 1. Introduction

Cannabis, including *Cannabis sativa* and *Cannabis indica*, originated in the Altai Mountains in southern Siberia, Russia, and has been widely used in everyday items, such as textiles, as well as in the medical and pharmaceutical fields. The earliest record of its medical use dates back to China 5000 years ago. In the context of modern medicine, the first use of cannabis was reported in the 1830s as an effective treatment for patients with mental illnesses [1,2]; however, further research revealed that it exhibits clear narcotic effects. The Cannabis Control Act, which was enacted in Japan in 1948, completely banned the possession and use of cannabis. The Δ^9^-THC content of oils/powders, liquids, and other forms of each product is limited to maxima of 0.001%, 0.00001%, and 0.0001%, respectively [3]. Despite this, the number of people arrested for cannabis-related crimes is currently increasing. According to a report from the Japanese Ministry of Health, Labor, and Welfare, about 70% of those arrested in recent years are under 30 years of age, with the younger age of arrest having become a social problem. In addition, more than 850 kg of dried cannabis was seized in 2023, and the number of synthetic drugs is also increasing yearly; consequently, the number of illegal drug appraisals is also increasing, which places pressure on the appraisal system [4]. Normally, expensive, highly technical, and time-consuming techniques, such as gas chromatography (GC), liquid chromatography/mass spectrometry (LC/MS), and high-performance liquid chromatography (HPLC), are required to accurately analyze cannabis. Therefore, the ability to determine the presence of narcotics or illegal substances prior to conducting any detailed analysis and to screen seized items before conducting an accurate analysis will lead to improved forensic work and a higher number of cannabis-related arrests.

**Figure 1:**
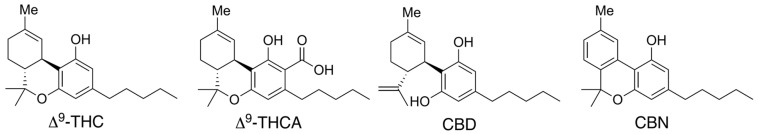
Structures of cannabinoids.

Cannabinoids are the main components of cannabis (Figure 1), with more than 100 types reported to date. Among them, Δ^9^-tetrahydrocannabinol (Δ^9^-THC) is a psychoactive narcotic, which exists in cannabis in the form of a carboxylic acid (Δ^9^-THCA) that is decarboxylated by heat or light to produce Δ^9^-THC [5]. In contrast, cannabidiol (CBD) is not psychoactive. Furthermore, many recreational products containing CBD are available owing to their relaxation effects. CBD has also been shown to have medicinal effects, including analgesic properties, and has been studied as a treatment for epilepsy [6,7,8]. Cannabinol (CBN), an analog produced by the oxidation of Δ^9^-THC, is very weakly psychoactive [9]. Consequently, numerous CBD- and CBN-containing products are available. As useful cannabinoids that do not show psychoactive properties exist, distinguishing Δ^9^-THC, which is psychoactive, from CBD and CBN, which are not, is important to prevent drug abuse.

**Figure 2:**
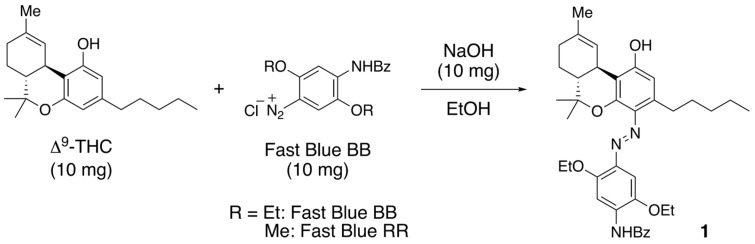
Diazo-coupling reaction and the reported colored product from Δ^9^-THC and Fast Blue BB.

Color-based reactions have been used to detect Δ^9^-THC; these classic methods determine the presence of Δ^9^-THC by the color of the compound produced by reacting a color-inducing reagent with the cannabinoid. Various such reactions exist, the most well-known of which are the Beam, Ghamrawy, Duquenois, and diazo-coupling reactions [10]. The Beam reaction is the oldest color-based reaction for cannabis. Here, a 5% ethanolic solution of potassium hydroxide is added to a cannabis extract to produce a deep purple color [11]. However, this reaction only gives a positive result in response to analogs bearing two free phenolic hydroxy groups, such as CBD, which is disadvantageous [12,13]. The substance responsible for this color is presumed to be a paraquinone, which is the oxidation product of CBD [14]. The Ghamrawy reagent, which uses a concentrated sulfuric acid solution of *p*-dimethylaminobenzaldehyde, turns reddish brown in response to most cannabinoids and then blue when the solution is diluted with water [15]. Compounds that are not cannabinoids can also trigger a color change; however, they can be distinguished owing to differences in color tone. Unfortunately, this reaction cannot distinguish carboxylic acids, such as Δ^9^-THCA. The product of this reaction is believed to be a polymethylene carbonium ion formed by the combination of the cannabinoid and the Ghamrawy reagent [16]. The Duquenois reaction is widely used to identify cannabis. This reagent is an ethanolic solution of acetaldehyde and vanillin. The cannabis extract and concentrated hydrochloric acid are added to the Duquenois reagent in this method; the extract is initially sea-green in appearance but gradually changes to blue and then purple [17]. However, while this reagent responds in a similar manner to herbal medicines other than cannabis, it can still be used to distinguish them. The substance responsible for the generated color is believed to be a polymethylene carbonium ion formed by the combination of the cannabinoid and the Duquenois reagent in a similar manner to its combination with the Ghamrawy reagent [18]. The diazo-coupling reaction has also been widely used as a color-based reaction for detecting cannabis, despite being first used as a thin-layer chromatography (TLC) reagent because the reaction of a cannabinoid with Fast Blue BB (a diazonium salt) is highly sensitive [19]. The method was refined to selectively detect cannabinoids rather than herbal products and natural medicines, which resulted in a simplified method for on-site cannabis detection. The substance responsible for the observed color is presumed to be the azo compound formed by reacting the cannabinoid with the diazonium compound. In 2020, Almirall et al. isolated and determined the structure of product 1 from the reaction of Δ^9^-THC with Fast Blue BB (Figure 2) [20]. However, the structures of other cannabinoid products from this reaction remain undetermined.

As discussed above, while various color-based reactions have been used to detect cannabis, the substances responsible for the observed colors have hardly been studied, with many structures remaining speculative. To address this issue, details of the products obtained from the diazo-coupling reaction have been determined by isolating and determining the structures of the reaction products from three cannabinoids, namely CBD, CBN, and Δ^9^-THC, with the Fast Blue RR diazonium salt. Fast Blue RR is an analog of Fast Blue BB with a different ether substituent. Although there is almost no difference in their reactivities, structural analysis using Fast Blue RR is easier because it has a simple methyl group.

## 2. Results

### 2.1. Colored Product **2** from the Reaction of CBD with Fast Blue RR

**Figure 3:**
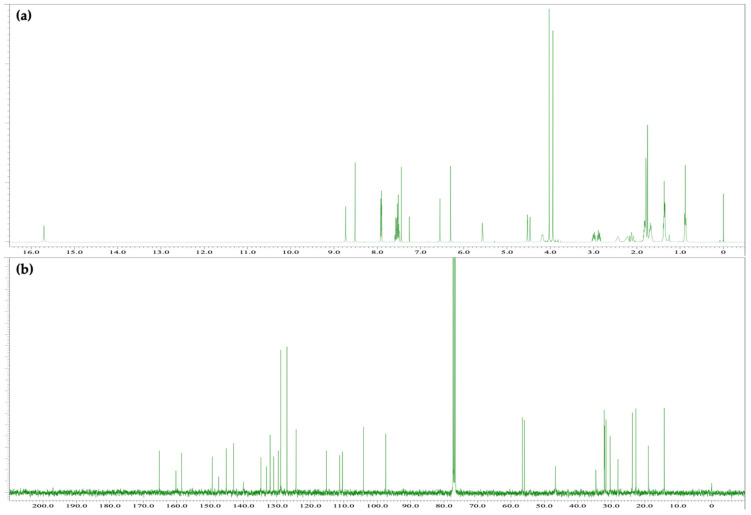
(**a**) ^1^H NMR [400 MHz, CDCl_3_] and (**b**) ^13^C NMR [100 MHz, CDCl_3_] spectra of **2**.

**Figure 4:**
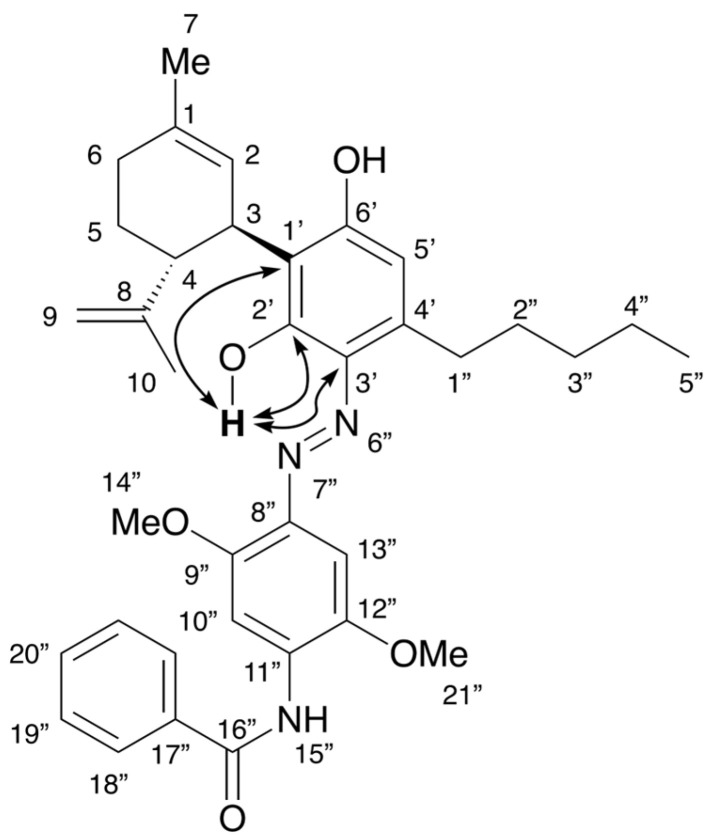
Key HMBC correlations for **2**.

The reaction between CBD and Fast Blue RR was carried out in an ethanolic NaOH solution, which resulted in a red mixture. TLC revealed that CBD disappeared after 15 min and several new spots formed. Product **2** corresponded to an orange spot with an *R*_f_ value of 0.59 (hexane/ethyl acetate = 1/3) that was subsequently isolated by silica-gel column chromatography. Figure 3 shows the ^1^H and ^13^C NMR spectra of **2**. The ^1^H NMR spectrum of **2** showed 43 proton signals, with integration showing that **2** is formed by the reaction of CBD with 1 equiv of Fast Blue RR. This is also supported by the high-resolution mass spectra (HRMS) (See Section 4. Materials and Methods), in which the downfield peak at 15.75 ppm corresponds to a proton on either an oxygen or a nitrogen atom. Heteronuclear multiple bond connectivity (HMBC) correlations were also observed between this proton signal and the carbon peaks at 158.5, 131.0, and 115.2 ppm (Figure 4 and Figure S5). Since the peak at 115.2 ppm is assigned to the carbon at the 1′ position, the proton signal at 15.75 ppm corresponds to the proton of the phenol bonded to the benzene ring derived from CBD. This peak is assigned to the hydroxy group at the 2′ position because it can form a hydrogen bond with the lone pair on the nitrogen atom of the azo group. Accordingly, **2** was determined to be an azo compound bearing two phenolic moieties and was isolated in 60% yield (Table 1). The other spots observed by TLC correspond to oxidized CBD (i.e., a benzoquinone), decomposed Fast Blue RR, and trace amounts of the bi-azo compound derived from CBD.

### 2.2. Colored Product **3** from the Reaction of CBN with Fast Blue RR

**Figure 5:**
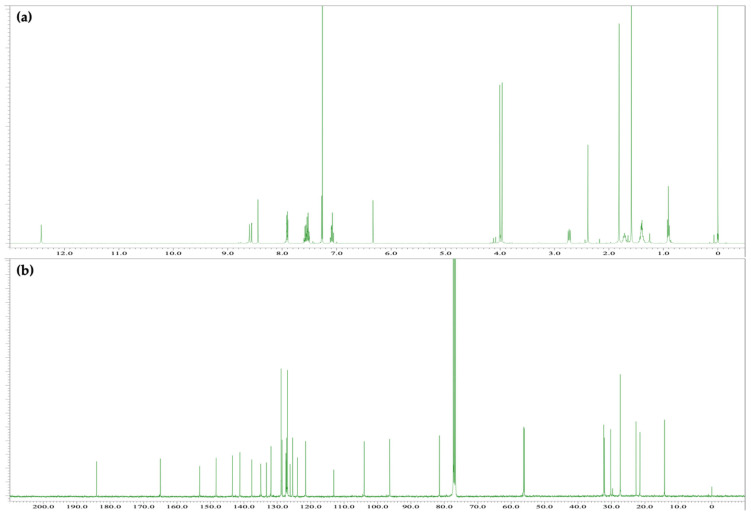
(**a**) ^1^H NMR [400 MHz, CDCl_3_] and (**b**) ^13^C NMR [100 MHz, CDCl_3_] spectra of **3**.

**Figure 6:**
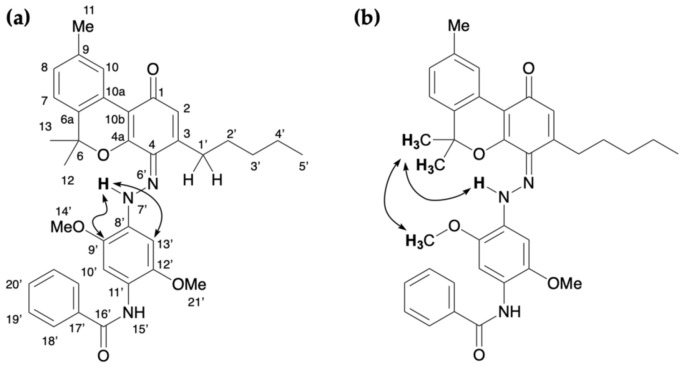
Key correlations for **3**: (**a**) HMBC and (**b**) NOESY.

The reaction of CBN and Fast Blue RR was carried out in an ethanolic NaOH solution in a similar manner to the reaction of CBD to produce a red mixture. The spot corresponding to CBN diminished after 15 min, and a new large spot was observed by TLC, along with a spot corresponding to decomposed Fast Blue RR. The main purple spot had an *R*_f_ value of 0.32 (hexane/ethyl acetate = 1/3) and was isolated by silica-gel column chromatography to afford compound **3**, whose ^1^H and ^13^C NMR spectra are displayed in Figure 5. The ^1^H NMR spectrum of **3** showed 39 proton signals. Integration and HRMS revealed that **3** is the product of the reaction of CBN with 1 equiv of Fast Blue RR, as was observed for **2**. In addition, a signal for a proton on an oxygen or nitrogen atom was observed at 12.43 ppm; this chemical shift is at a higher magnetic field than the analogous peak observed for **2**. The ^13^C NMR spectrum of **3** showed a peak at 184.0 ppm, which is characteristic of a conjugated carbonyl group. Since this chemical shift is unusual for the proposed azo compound (i.e., the analog of **1**), the two-dimensional NMR spectra of **3** were analyzed, which revealed HMBC correlations between the proton signal at 12.43 ppm and the carbon peaks at 141.2 and 96.3 ppm (Figure 6a and Figure S11). Since these carbons are assigned to the benzene ring of the Fast Blue RR moiety, the proton signal at 12.43 ppm is unlikely to correspond to a phenolic proton. Taken together, these results suggest that **3** is a quinoneimine rather than an azo compound; accordingly, **3** contains an oxidized benzene ring. Furthermore, the methyl signal of the pyran ring and the methoxy signal of the Fast Blue RR moiety are correlated by nuclear Overhauser effect spectroscopy (NOESY) (Figure 6b and Figure S12), which indicates that the Fast Blue RR moiety is bonded to the 4-position of CBN. In other words, the product of the reaction between CBN and Fast Blue RR is a quinoneimine rather than an azo compound, as previously proposed (Table 2). Compound **3** was isolated in 64% yield.

### 2.3. Colored Product **4** from the Reaction of Δ^9^-THC with Fast Blue RR

**Figure 7:**
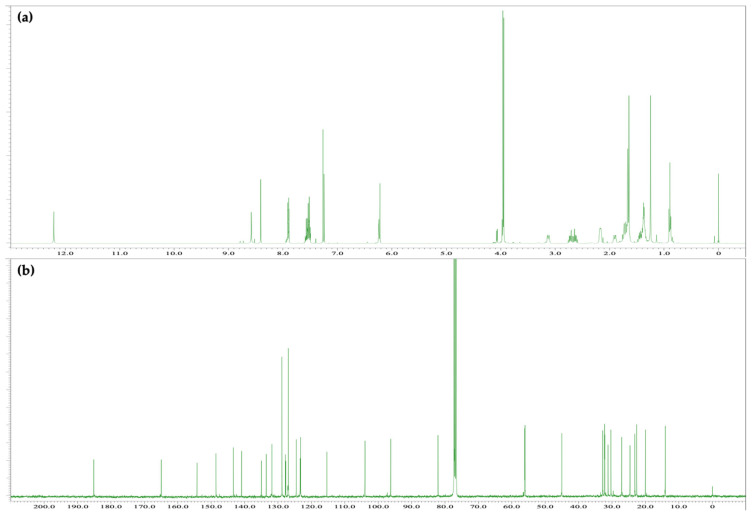
(**a**) ^1^H NMR [400 MHz, CDCl_3_] and (**b**) ^13^C NMR [100 MHz, CDCl_3_] spectra of **4**.

**Figure 8:**
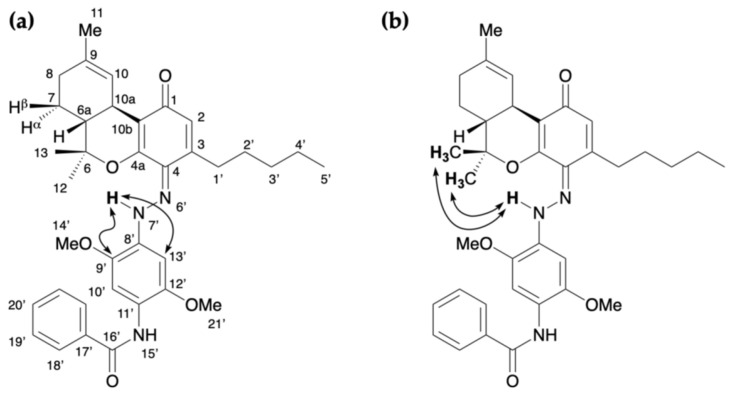
Key correlations for **4**: (**a**) HMBC and (**b**) NOESY.

The reaction between Δ^9^-THC and Fast Blue RR was carried out in an ethanolic NaOH solution, as described above, to afford a red reaction mixture. The spot corresponding to Δ^9^-THC disappeared after 15 min, and several new spots were observed by TLC. Although decomposition products from Fast Blue RR were observed, the main red spot exhibited an *R*_f_ value of 0.22 (hexane/ethyl acetate = 1/3) and was subsequently isolated by silica-gel column chromatography to afford compound **4**, whose ^1^H and ^13^C NMR spectra are displayed in Figure 7. The ^1^H NMR spectrum of **4** showed 43 signals. Integration and HRMS revealed that **4** is a product of the reaction of Δ^9^-THC with 1 equiv of Fast Blue RR, as was the case for compounds **2** and **3**. In addition, a signal was observed at 12.21 ppm in the ^1^H NMR spectrum of **4**, which corresponds to a proton on an oxygen or nitrogen atom, in a similar manner to the downfield proton signal observed for **3**, the product of the reaction of CBN with Fast Blue RR. Meanwhile, a peak at 185.2 ppm, which is characteristic of a conjugated carbonyl carbon, was observed in the ^13^C NMR spectrum. HMBC correlations between the proton signal at 12.21 ppm and the carbon signals at 140.9 and 96.3 ppm, which are assigned to the benzene ring of the Fast Blue RR moiety, were observed (Figure 8a and Figure S17). Furthermore, NOESY correlations were observed between the proton signal at 12.21 ppm and the signals for the methyl groups on the dimethylpyran ring (Figure 8b and Figure S18). Taken together, these results suggest that compound **4** contains a Fast Blue RR moiety bonded via a quinoneimine linkage to the position *para* to the original phenolic group in Δ^9^-THC, in a similar manner to that in compound **3**. In other words, the compound formed by the reaction of Δ^9^-THC with the Fast Blue RR diazonium salt (or Fast Blue BB by inference) is a quinoneimine rather than an azo compound, in contrast to the previously proposed structure (Table 3). Compound **3** was isolated in 60% yield; the other spots observed by TLC are attributable to products arising from the decomposition of Δ^9^-THC and **4**.

## 3. Discussion

**Figure 9:**
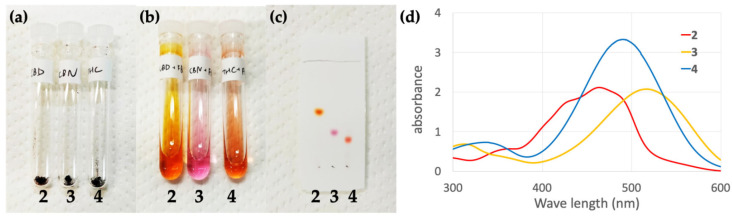
Photographic images of **2**–**4**: (**a**) in powder form, (**b**) in dilute solution, (**c**) and on a TLC plate. (**d**) UV-vis spectra of **2**–**4**: 2 (red), **3** (yellow), and **4** (blue).

Isolated compounds **2**–**4** are black powders (Figure 9a). Azo compound **2** forms an orange solution when dissolved in ethanol at low concentrations, such as that used for TLC, while quinoneimines **3** and **4** produced purple and red solutions, respectively; these colors enabled these compounds to be distinguished both in solution and on the TLC plate (Figure 9b,c). Furthermore, the findings are in good agreement with those of a detailed color discrimination of TLC between cannabinoids and Fast Blue RR by Duarte-Almeida et al. [21]. In addition, 0.1 mM ethanol solutions of compounds **2**–**4** were prepared, and their UV-vis spectra were acquired (Figure 9d). The three compounds exhibited different absorption maxima: 420 nm for azo compound **2** and 445 and 435 nm for quinoneimines **3** and **4**, respectively. These results show that pure products **2**–**4** are distinguishable by color. Because the isolated, highly pure compounds exhibit distinct colors, the hypothesis was created that the main components of cannabis can be identified by color if more reactive diazonium salts were used. However, the cannabis plant contains numerous cannabinoids. For example, Tanaka et al. reported that in addition to the main component Δ^9^-THCA (5.1–10.5 µg/mg), drug-type leaves contained CBDA (0.02 µg/mg), while fiber-type leaves, in which CBDA is the main component (1.5–3.3 µg/mg), contained Δ^9^-THCA (0.1 µg/mg) [22]. In addition to cannabinoids, terpenes and flavonoids have been isolated from cannabis plants [23]. Compounds with phenolic hydroxyl groups are susceptible to the diazo coupling reaction; therefore, multiple cannabinoids and flavonoids present in the cannabis plant may produce color. False positives, which are a major problem during the actual detection of cannabis, are believed to be caused by the products of other cannabis components reacting with the diazonium salt used in the color-based reaction. The results of this study suggest that, in addition to developing reagents with better color-discriminating capabilities, research on reagents that are highly reactive toward cannabinoids may also lead to the development of better color reagents.

**Figure 10:**
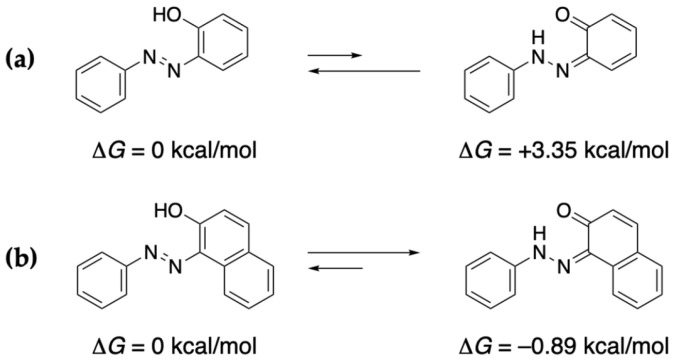
Azo–quinoneimine tautomerism: (**a**) azobenzene derivative (**b**) naphthalene derivative.

The structure of the product isolated from the reaction of Δ^9^-THC with the diazonium salt differs from that reported in the literature [20]. A quinoneimine is thought to be formed owing to stability considerations. The structure obtained is determined by the energetics of the system; specifically, how the structure is stabilized through resonance associated with an azo-type compound versus hydrogen bonding in a quinoneimine-type compound. In a related study, Özen et al. calculated the stabilities of these compound types [24]. The azo-type structure is more stable in the case of the azobenzene derivative (Figure 10a), whereas the quinoneimine-type structure is more stable in the case of the naphthalene derivative (Figure 10b). In other words, resonance stabilization is expected to play a major role in determining the product formed from CBD, whereas stabilization by hydrogen bonding plays a major role for CBN and Δ^9^-THC. Therefore, the stability of the compound produced by the color reaction also affects its color.

The azo–quinoneimine tautomerism is also believed to be affected by the solvent and pH. In relation to this, Karci et al. examined the azo–quinoneimine tautomerism using a compound featuring an azo group at the 3-position of 2-hydroxy-1,4-naphthoquinone, which revealed that its color is influenced by basicity [25]. However, the effect of the solvent could not be clarified. In the future, we plan to investigate the tautomerism and color changes associated with compounds **2**–**4** in response to pH and in various solvents to further clarify their characteristics, which is expected to contribute to the development of color-based reagents for better cannabis detection.

## 4. Materials and Methods

### 4.1. Materials

CBD powder was purchased from CannaTech Co., Ltd. (Kanagawa, Japan), CBN powder was purchased from Leep Co., Ltd. (Tokyo, Japan), and Fast Blue RR was purchased from Sigma–Aldrich Co., LLC (St. Louis, MO, USA). All compounds were used as received. Δ^9^-THC was synthesized from CBD using a previously reported method [26]; NMR data for the Δ^9^-THC prepared in this manner matched those previously reported [27]. Other reagents were purchased from commercial suppliers and used as received.

### 4.2. General Synthesis and Analysis Information

Reaction mixtures were magnetically stirred and monitored by TLC using pre-coated silica-gel plates. Column chromatography was performed using silica gel (60 μm). ^1^H (400 MHz) and ^13^C (100 MHz) NMR spectra were recorded on a JNM-ECZ400S NMR spectrometer (JEOL Ltd., Tokyo, Japan) at 296 K unless otherwise stated. Chemical shifts are given in parts per million (ppm) downfield from that of tetramethylsilane as an internal standard (0.00 ppm), and coupling constants (*J*) are reported in Hz. Splitting patterns are abbreviated as: singlet (s), doublet (d), triplet (t), multiplet (m), and broad (br). High-resolution mass spectra (HRMS) were recorded on an X500R QTOF electrospray ionization time-of-flight mass spectrometer (AB Sciex LLC, Marlborough, MA, USA). IR spectra were recorded on a Spectrum 100 FT-IR spectrometer equipped with an ATR (diamond) accessory (PerkinElmer, Inc., Waltham, MA, USA). UV-vis spectra were obtained using ethanol solutions of the samples (0.1 mM) and recorded on a V-750 Spectrophotometer (JASCO Corporation, Tokyo, Japan).

### 4.3. General Methods for Identifying Colored Products

Fast Blue RR (1 equiv) was added to a stirred solution of the cannabinoid (1 equiv) and NaOH (5 equiv) in EtOH (0.05 mol/L) at 23 °C. The mixture was stirred for 15 min, after which half the volume of solvent was removed in vacuo, water was added, and the pH was adjusted to 7 using 1 N HCl. The crude product was extracted with ethyl acetate (×3), and the combined organic extracts were washed with brine, dried with Na_2_SO_4_, and concentrated in vacuo. The residue was purified by flash column chromatography (hexane/ethyl acetate) to obtain the product.

### 4.4. Spectral Data

**2**: CBD (0.20 mmol scale), 60% yield; purple solid; *R*_f_ 0.59 (hexane/ethyl acetate = 1/3); ^1^H NMR (400 MHz, CDCl_3_) *δ* 15.71 (s, 1H, OH), 8.74 (s, 1H, NH), 8.52 (s, 1H), 7.93–7.91 (m, 2H), 7.61–7.51 (m, 3H), 7.47 (s, 1H), 6.53 (brs, 1H, OH), 6.31 (s, 1H), 5.58 (s, 1H), 4.53 (s, 1H), 4.46 (s, 1H), 4.22–4.16 (m, 1H), 4.04 (s, 3H), 3.96 (s, 3H), 3.04–2.97 (m, 1H), 2.91–2.84 (m, 1H), 2.48–2.40 (m, 1H), 2.30–2.20 (m, 1H), 2.13–2.08 (m, 1H), 1.85–1.80 (m, 2H), 1.80 (s, 3H), 1.75 (s, 3H), 1.71–1.66 (m, 2H), 1.41–1.34 (m, 4H), 0.88 (t, 3H, *J* = 6.8 Hz); ^13^C NMR (100 MHz, CDCl_3_) *δ* 165.2, 160.2, 158.5, 149.4, 147.4, 145.2, 142.9, 140.0, 134.8, 133.1, 132.0, 131.0, 129.6, 128.9, 127.0, 124.3, 115.2, 111.2, 110.4, 104.1, 97.5, 56.6, 56.0, 46.7, 34.6, 32.1, 32.0, 31.6, 30.3, 28.0, 23.7, 22.6, 18.9, 14.1; IR (ATR) 3416, 2924, 1677, 1522, 1466, 1251 cm^–1^; HRMS (ESI) *m*/*z* calcd. for C_36_H_44_N_3_O_5_ ([M + H]^+^): 598.3275, found: 598.3275.

**3**: CBN (0.20 mmol scale), 64% yield; purple solid; *R*_f_ 0.32 (hexane/ethyl acetate = 3/1); ^1^H NMR (400 MHz, CDCl_3_) *δ* 12.43 (s, 1H, NH), 8.60 (s, 1H, NH), 8.56 (d, 1H, *J* = 1.6 Hz), 8.44 (s, 1H), 7.92–7.90 (m, 2H), 7.60–7.50 (m, 3H), 7.28 (s, 1H), 7.15 (dd, 1H, *J* = 1.6, 8.0 Hz), 7.07 (d, 1H, *J* = 8.0 Hz), 6.33 (s, 1H), 4.00 (s, 3H), 3.96 (s, 3H), 2.75–2.71 (m, 2H), 2.39 (s, 3H), 1.82 (s, 6H), 1.75–1.68 (m, 2H), 1.44–1.37 (m, 4H), 0.91 (t, 3H, *J* = 7.2 Hz); ^13^C NMR (100 MHz, CDCl_3_) *δ* 184.0, 165.0, 153.2, 148.3, 143.4, 141.2, 137.6, 134.9, 133.2, 131.9, 128.8, 128.5, 127.4, 127.3, 127.2, 126.9, 126.1, 125.4, 124.0, 121.5, 113.1, 104.0, 96.3, 81.5, 56.2, 56.1, 32.3, 32.1, 30.2, 27.4, 22.6, 21.5, 14.1; IR (ATR) 3281, 2926, 1670, 1620, 1525, 1410, 1257 cm^–1^; HRMS (ESI) *m*/*z* calcd. for C_36_H_40_N_3_O_5_: 594.2962 (M + H)^+^, found: 594.2956.

**4**: Δ^9^-THC (50 µmol scale), 60% yield; purple solid; *R*_f_ 0.22 (hexane/ethyl acetate = 3/1); ^1^H NMR (400 MHz, CDCl_3_) *δ* 12.21 (s, 1H, NH), 8.58 (s, 1H, NH), 8.41 (s, 1H), 7.92–7.88 (m, 2H), 7.60–7.49 (m, 3H), 7.24 (s, 1H), 6.24 (t, 1H, *J* = 1.2 Hz), 6.22 (s, 1H), 3.96 (s, 3H), 3.95 (s, 3H), 3.16–3.09 (m, 1H), 2.77–2.59 (m, 2H), 2.21–2.14 (m, 2H), 1.94–1.87 (m, 1H), 1.76–1.63 (m, 3H), 1.67 (s, 3H), 1.65 (s, 3H), 1.50–1.33 (m, 5H), 1.25 (s, 3H), 0.89 (t, 3H, *J* = 7.2 Hz); ^13^C NMR (100 MHz, CDCl_3_) *δ* 185.2, 165.0, 154.3, 148.6, 143.4, 140.9, 135.0, 133.5, 131.8, 128.8, 127.8, 127.7, 126.9, 124.6, 123.4, 123.2, 115.4, 104.0, 96.3, 82.1, 56.2, 56.1, 45.1, 32.8, 32.24, 32.19, 31.2, 30.3, 27.1, 24.6, 23.2, 22.6, 20.0, 14.1; IR (ATR) 3287, 2926, 1667, 1620, 1513, 1411, 1252 cm^–1^; HRMS (ESI) *m*/*z* calcd. for C_36_H_44_N_3_O_5_: 598.3275 (M + H)^+^, found: 598.3275.

## 5. Conclusions

In this study, we examined the color-based diazo-coupling reaction used to detect cannabis and isolated products **2**–**4** from the reactions of the Fast Blue RR diazonium salt with three cannabinoids (CBD, CBN, and Δ^9^-THC). NMR-based structural elucidation revealed that product **2** obtained from CBD is an azo compound, whereas reaction products **3** and **4** obtained from CBN and Δ^9^-THC, respectively, are quinoneimines, in contrast to the compounds reported previously. The isolated compounds **2**–**4** could be distinguished by color. However, distinguishing the main cannabinoid from the other components of cannabis by color is still impossible because multiple cannabinoids and flavonoids present in the cannabis plant may produce color. To resolve this issue, highly sensitive color reagents are required to detect regulated compounds in mixtures with similar compositions. Research on the synthesis and development of such reagents is currently underway.

## Figures and Tables

**Table 1:** Assignment of the peaks in the ^1^H and ^13^C NMR spectra of **2**.

	^1^H	^13^C		^1^H	^13^C
1		140.0	1″	3.04−2.97 (m, 1H) 2.91−2.84 (m, 1H)	32
2	5.58 (s, 1H)	124.3	2″	1.71−1.66 (m, 2H)	31.6
3	4.22–4.16 (m, 1H)	34.6	3″	1.41−1.34 (m, 2H)	32
4	2.48–2.40 (m, 1H)	46.7	4″	1.41−1.34 (m, 2H)	22.6
5	1.85–1.80 (m, 2H)	28.0	5″	0.88 (t, 3H)	14.1
6	2.30–2.20 (m, 1H) 2.13–2.08 (m, 1H)	30.3	8″		133.1
7	1.80 (s, 3H)	23.7	9″		149.4
8		147.5	10″	8.52 (s, 1H)	104.1
9	4.53 (s, 1H) 4.46 (s, 1H)	111.2	11″		129.6
10	1.75 (s, 3H)	18.9	12″		142.9
1′		115.2	13″	7.47 (s, 1H)	97.5
2′		158.5	14″	4.04 (s, 3H)	56.6
2′-OH	15.71 (s, 1H)		15″-NH	8.74 (s, 1H)	
3′		131.0	16″		165.2
4′		145.1	17″		134.5
5′	6.31 (s, 1H)	110.4	18″	7.93−7.91 (m, 2H)	127.0
6′		160.2	19″	7.61−7.51 (m, 2H)	128.9
6′-OH	6.53 (brs, 1H)		20″	7.61−7.51 (m, 1H)	132.0
			21″	3.96 (s, 3H)	56.3

**Table 2:** Assignment of the peaks in the ^1^H and ^13^C NMR spectra of **3**.

	^1^H	^13^C		^1^H	^13^C
1		184.0	3′	1.44−1.33 (m, 2H)	32
2	6.33 (s, 1H)	125.4	4′	1.44−1.33 (m, 2H)	22.6
3		127	5′	0.91 (t, 3H)	14.1
4		148.3	7′-NH	12.43 (s, 1H)	
4a		153.2	8′		127
6		81.5	9′		141.2
6a		133.2	10′	8.44 (s, 1H)	104.0
7	7.07 (d, 1H)	121.5	11′		124.0
8	7.15 (dd, 1H)	128.5	12′		143.4
9		137.6	13′	7.28 (s, 1H)	96.3
10	8.56 (d, 1H)	127	14′	4.00 (s, 3H)	56
10a		126.1	15′-NH	8.60 (s, 1H)	
10b		113.1	16′		165.0
11	2.39 (s, 3H)	21.5	17′		134.9
12, 13	1.82 (s, 6H)	27.4	18′	7.92−7.90 (m, 2H)	126.9
1′	2.75–2.71 (m, 2H)	32	19′	7.60−7.50 (m, 2H)	128.8
2′	1.75–1.68 (m, 2H)	30.2	20′	7.60−7.50 (m, 1H)	131.9
			21′	3.96 (s, 3H)	56

**Table 3:** Assignment of the peaks in the ^1^H and ^13^C NMR spectra of **4**.

	^1^H	^13^C		^1^H	^13^C
1		185.2	3′	1.42−1.33 (m, 2H)	32
2	6.22 (s, 1H)	124.6	4′	1.42−1.33 (m, 2H)	22.6
3		127	5′	0.89 (t, 3H)	14.1
4		148.6	7′-NH	12.21 (s, 1H)	
4a		154.3	8′		127
6		82.1	9′		140.9
6a	1.76−1.63 (m, 1H)	45.1	10′	8.41 (s, 1H)	104.0
7	1.50−1.42 (m, 1H, 7a) 1.94−1.87 (m, 1H, 7b)	24.6	11′		123
8	2.21−2.14 (m, 2H)	31.2	12′		143.4
9		133.5	13′	7.24 (s, 1H)	96.3
10	6.24 (t, 1H)	123	14′	3.96 (s, 3H)	56
10a	3.16−3.09 (m, 1H)	32.8	15′-NH	8.58 (s, 1H)	
10b		115.4	16′		165.0
11	1.67 (s, 3H)	23.2	17′		135.0
12	1.25 (s, 3H)	20.0	18′	7.92−7.88 (m, 2H)	126.9
13	1.65 (s, 3H)	27.1	19′	7.60−7.49 (m, 2H)	128.8
1′	2.77−2.59 (m, 2H)	32	20′	7.60−7.49 (m, 1H)	131.8
2′	1.76−1.63 (m, 2H)	30.3	21′	3.95 (s, 3H)	56

## Data Availability

Data are contained within the article and Supplementary Materials.

## Supplementary Materials

The following supporting information can be downloaded at https://www.mdpi.com/article/10.3390/molecules30173462/s1, Figures S1–S6: NMR spectra of compound **2**; Figures S7–S12: NMR spectra of compound **3**; Figures S13–S18: NMR spectra of compound **3**.
