# Supplementary material for: Isolating and Determining the Structures of Colored Products from the Reactions of Cannabinoids with Fast Blue RR
Source: Molecules. 2025 Aug 22;30(17):3462. doi: 10.3390/molecules30173462 (PMC12430052; doi:10.3390/molecules30173462)

Supporting Information

Isolating and Determining the Structure of Colored Products from the Reaction of Cannabinoids with Fast Blue RR

Kayo Nakamura [1,*], Hikari Nishiguchi [1], Ryosuke Arai [1], Riho Hamajima [1], Hiroko Abe [2], Akihiko Ishida [3], Manabu Tokeshi [3], Kyohei Higashi [1], Akiyoshi Saitoh [1] and Hideyo Takahashi [1,*]

[1] Faculty of Pharmaceutical Sciences, Tokyo University of Science, 6-3-1 Niijuku, Katsushika-ku, Tokyo 125-8585, Japan

[2] BioDesign Inc., IB Daiichi-Bld 6th floor, 3-25-15 Nishi-Ikebukuro, Toshima-ku, Tokyo 171-0021, Japan

[3] Faculty of Engineering, Hokkaido University, Kita 13, Nishi 8, Kita-ku, Sapporo 060-8628, Japan

kayo_nakamura@rs.tus.ac.jp, hide-tak@rs.tus.ac.jp

Contents

1. NMR Spectra of compound **2**...S2
2. NMR Spectra of compound **3**...S8
3. NMR Spectra of compound **4**...S15

1. NMR Spectra of **2**

Figure S1. ^{1}H NMR (400 MHz, $CDCl_3$, ppm) of **2**

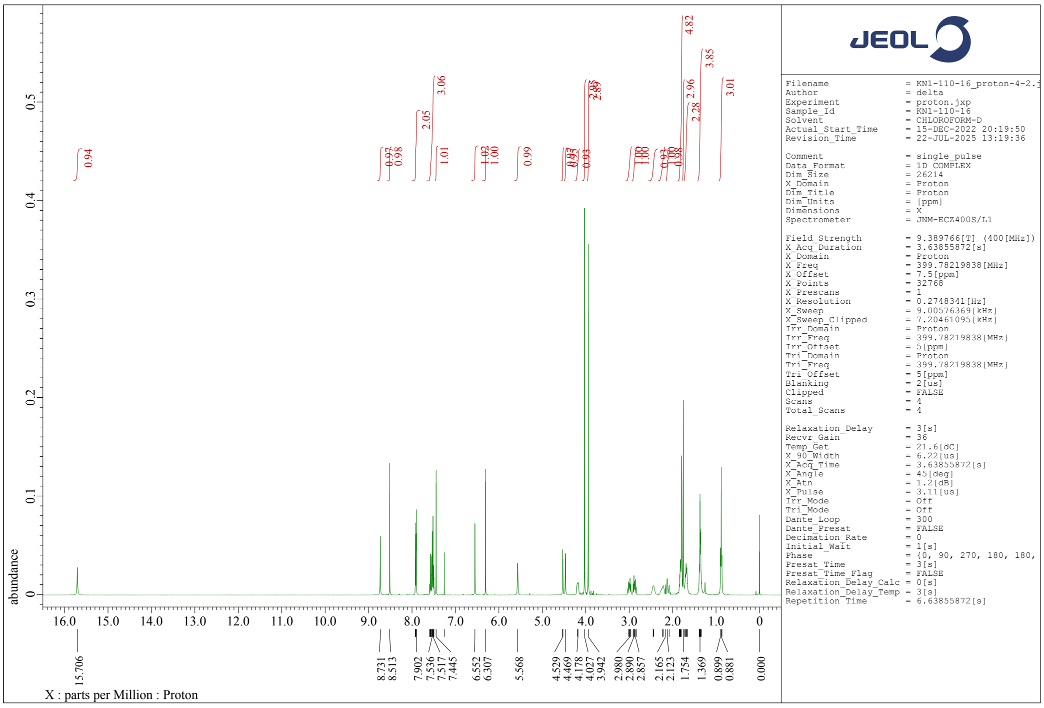

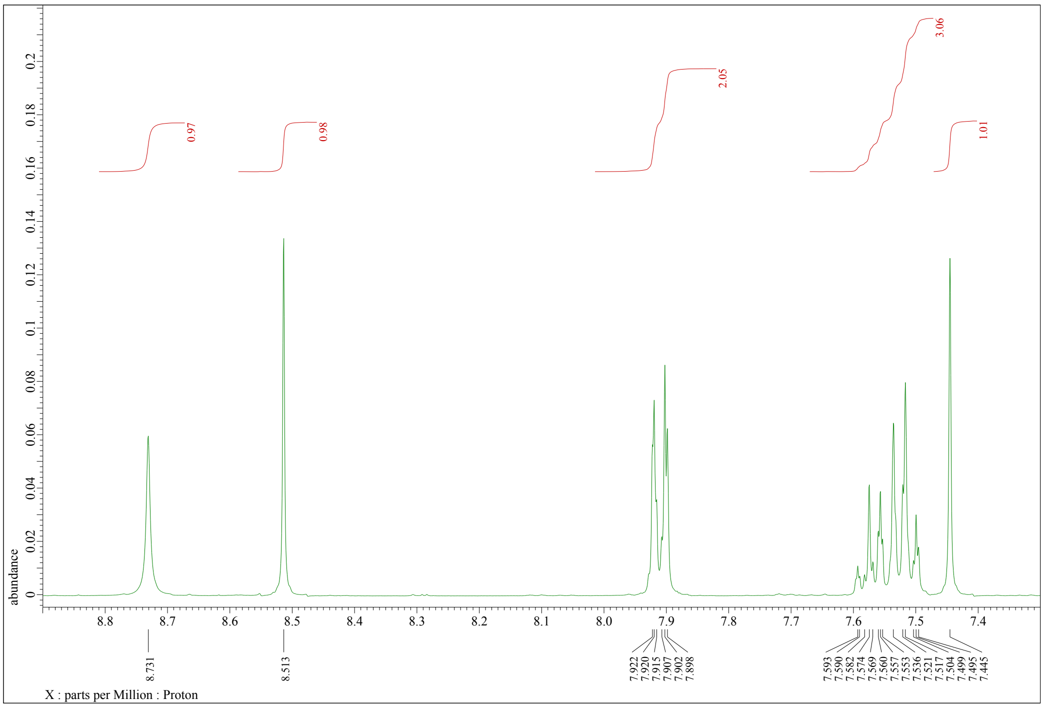

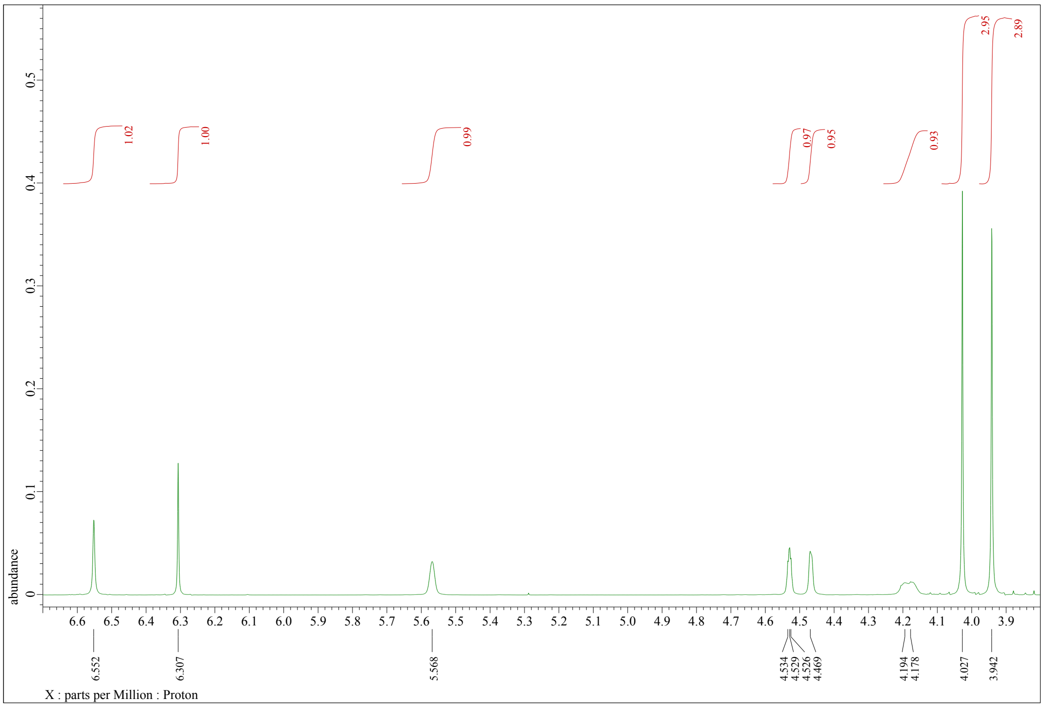

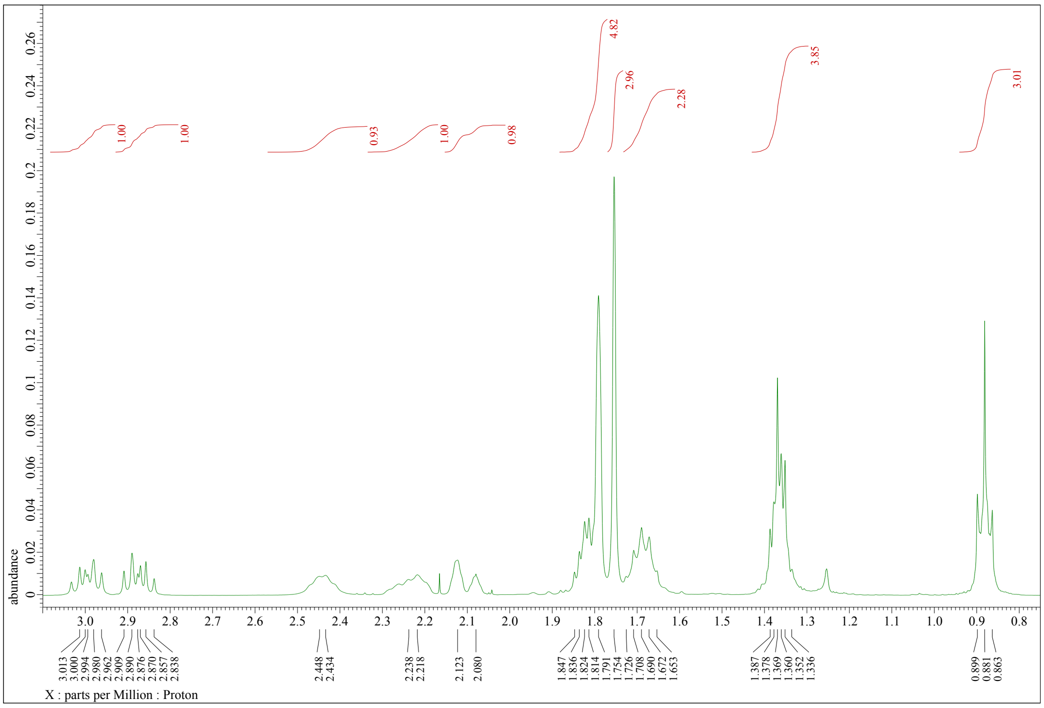

Figure S2. ^{13}C NMR (100 MHz, $CDCl_3$, ppm) of **2**

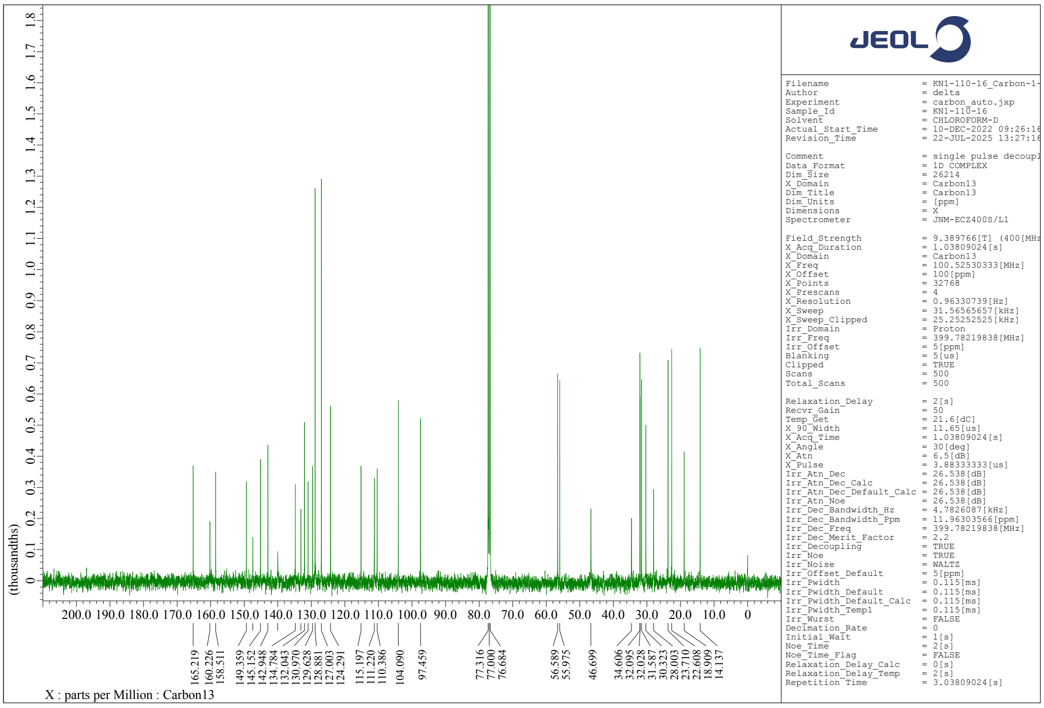

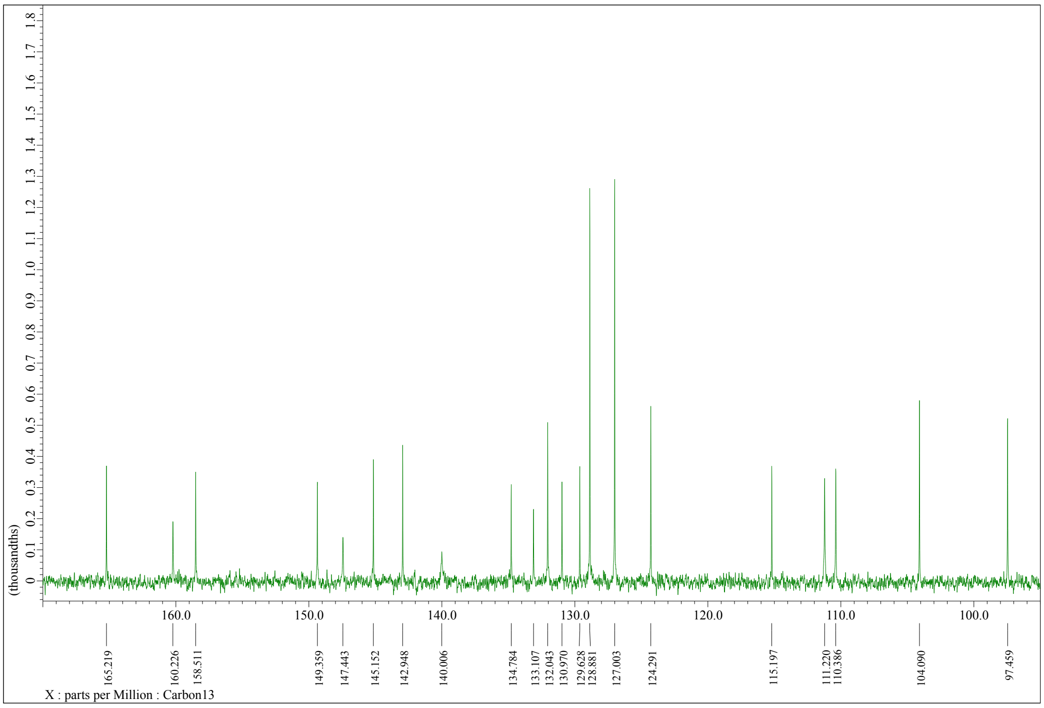

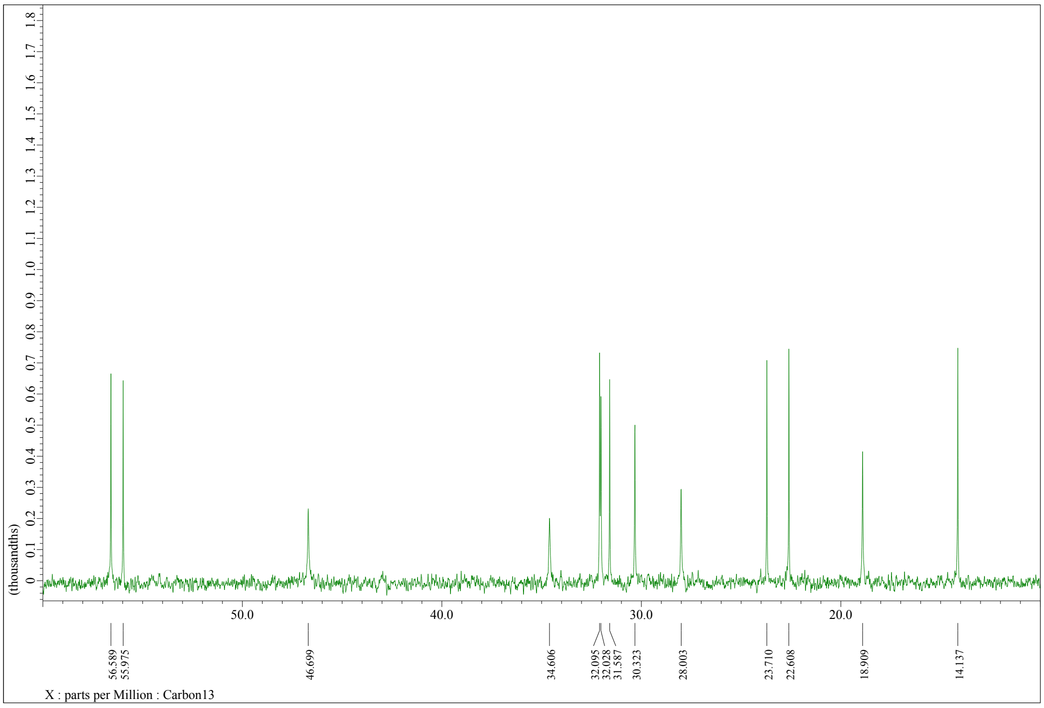

Figure S3. COSY spectrum of **2**

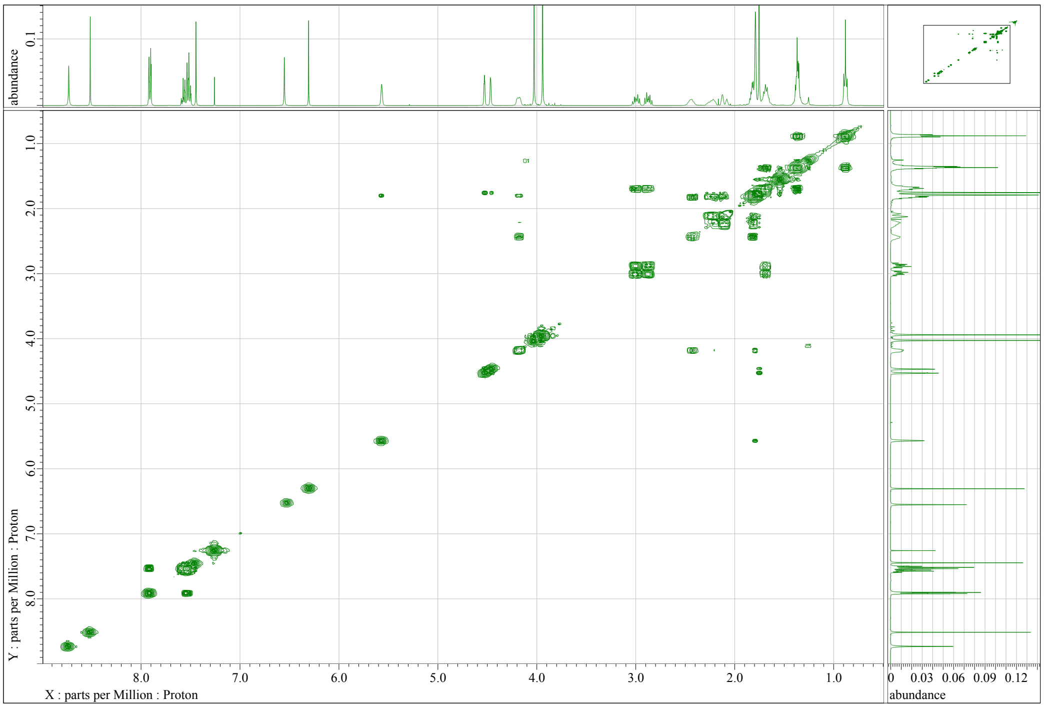

Figure S4. HMQC spectrum of **2**

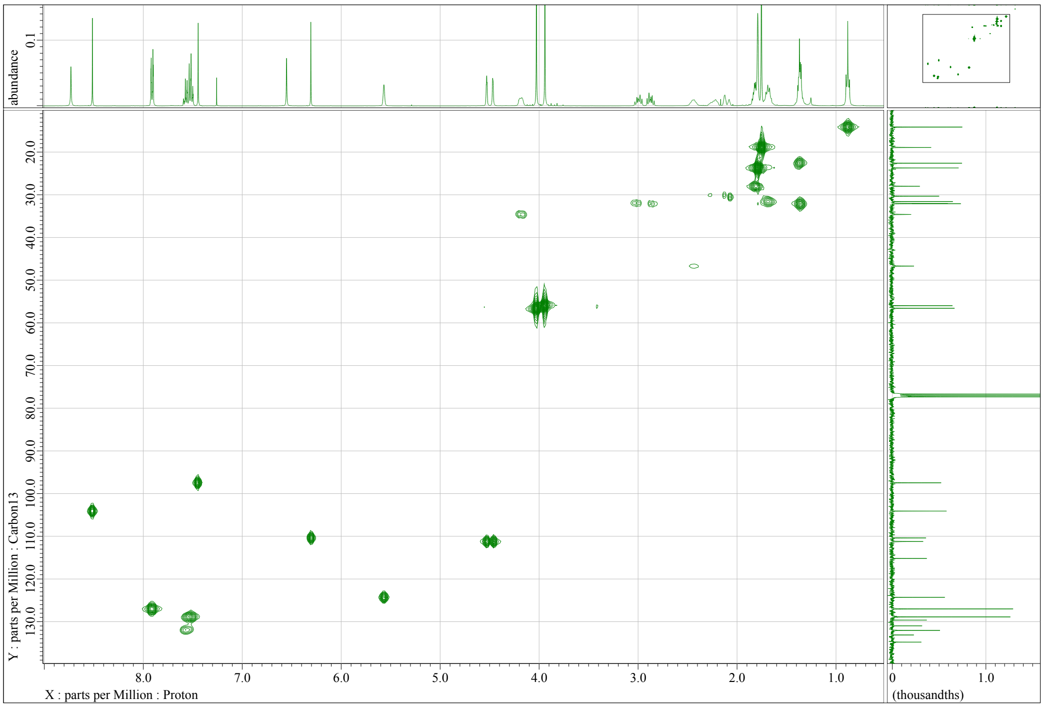

Figure S5. HMBC spectrum of **2**

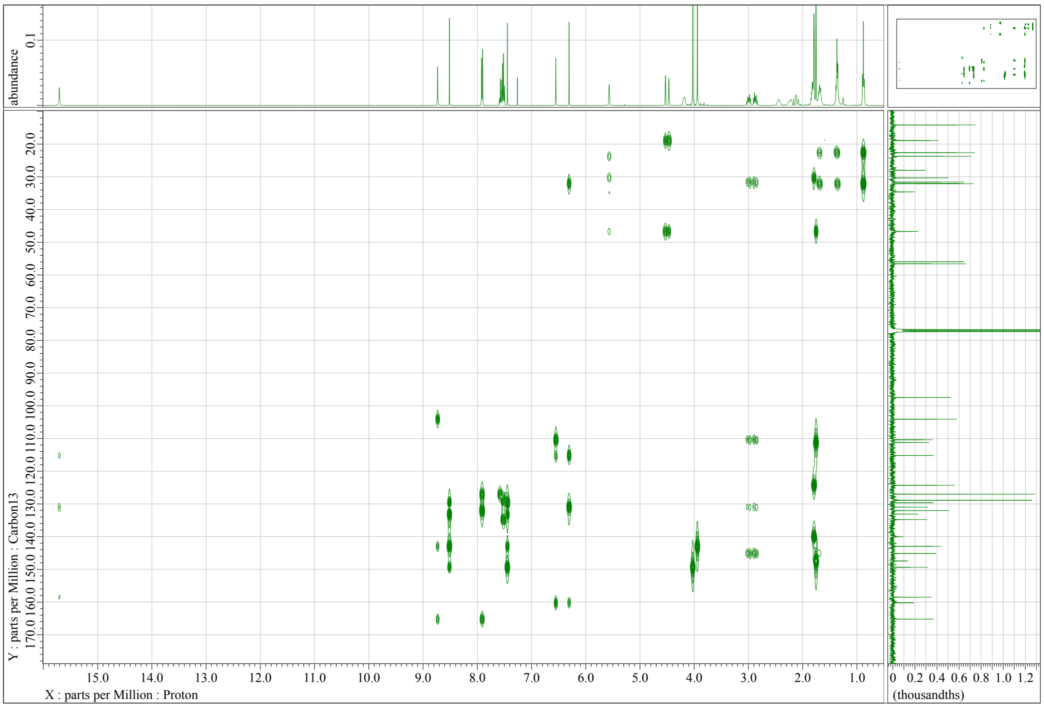

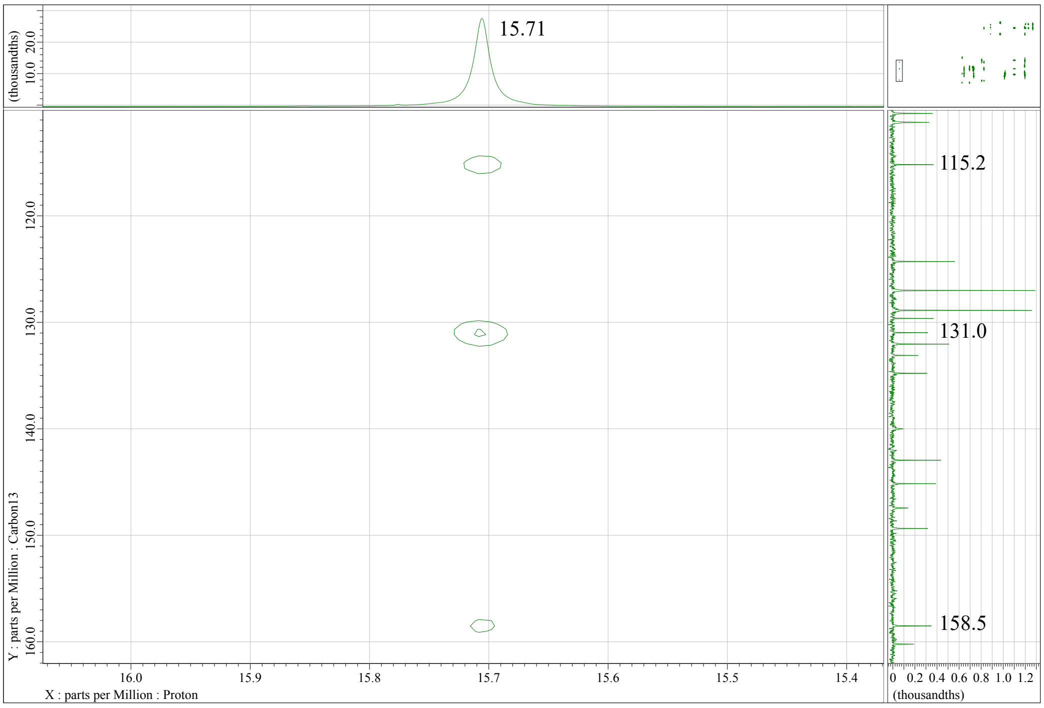

Figure S6. NOESY spectrum of **2**

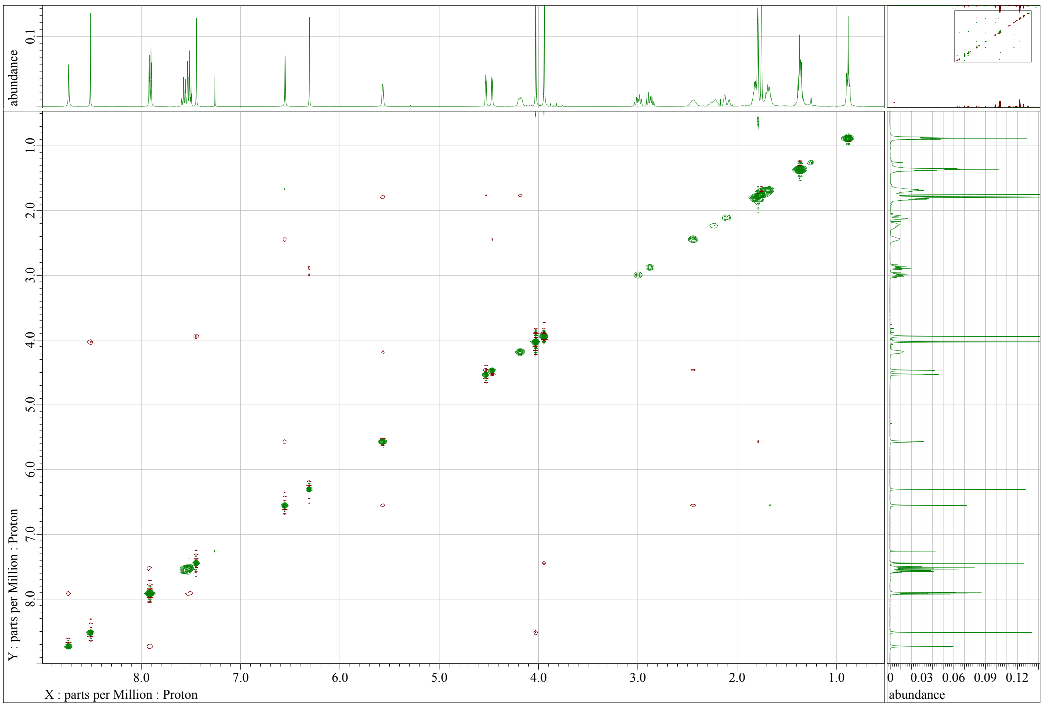

2. NMR Spectra of **3**

Figure S7. ^{1}H NMR (400 MHz, $CDCl_3$, ppm) of **3**

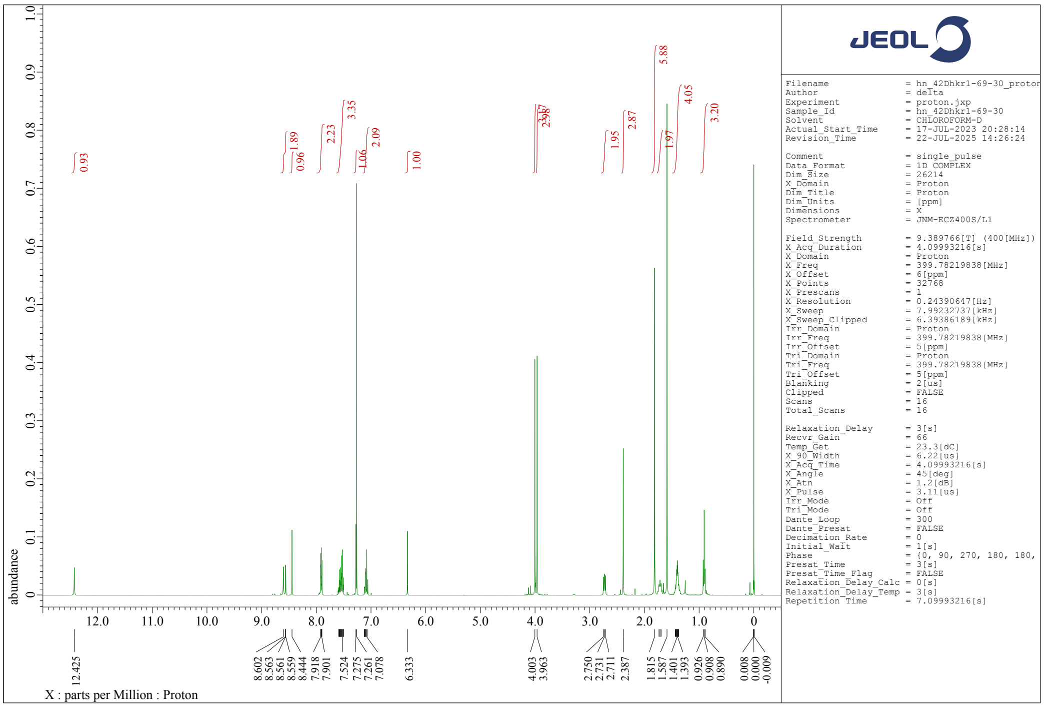

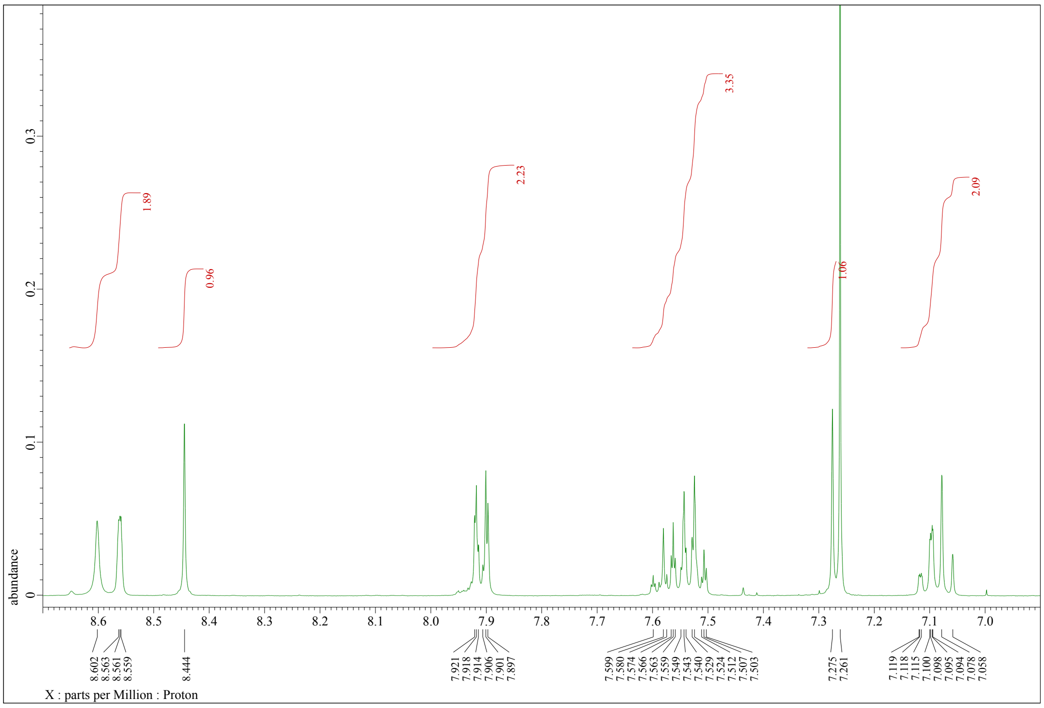

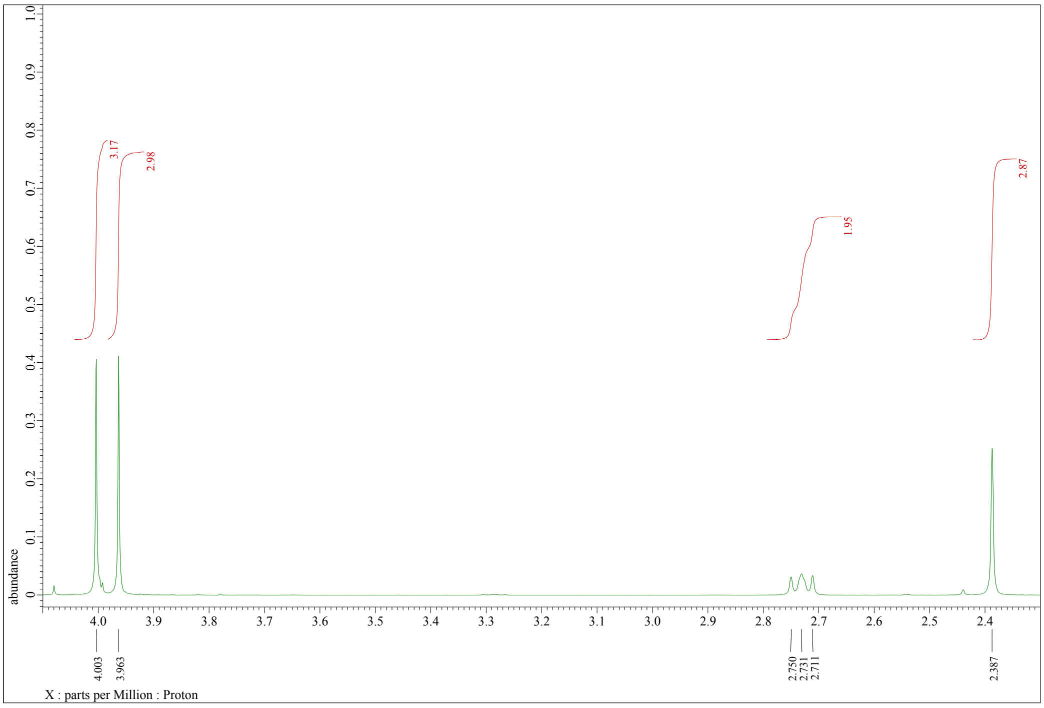
abundance
X : parts per Million : Proton
3.17
2.98
1.95
2.87
4.003
3.963
2.750
2.731
2.711
2.387

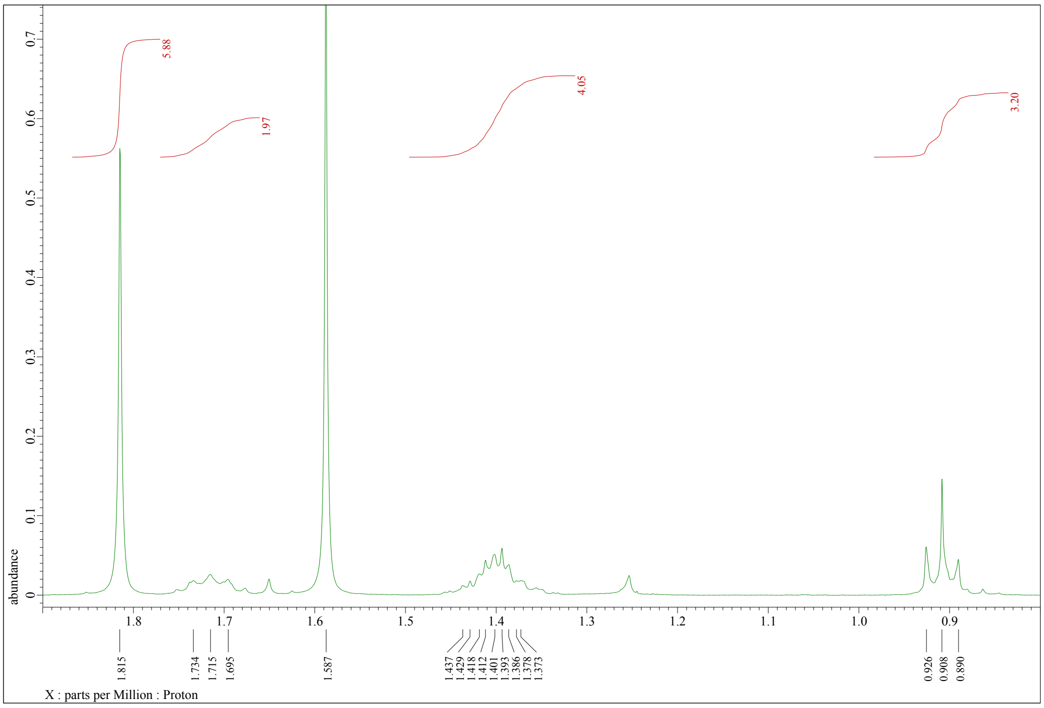
abundance
X : parts per Million : Proton
5.88
1.97
4.05
3.20
1.815
1.734
1.715
1.695
1.587
1.437
1.429
1.418
1.412
1.401
1.393
1.386
1.378
1.373
0.926
0.908
0.890

Figure S8. ^{13}C NMR (100 MHz, $CDCl_3$, ppm) of **3**

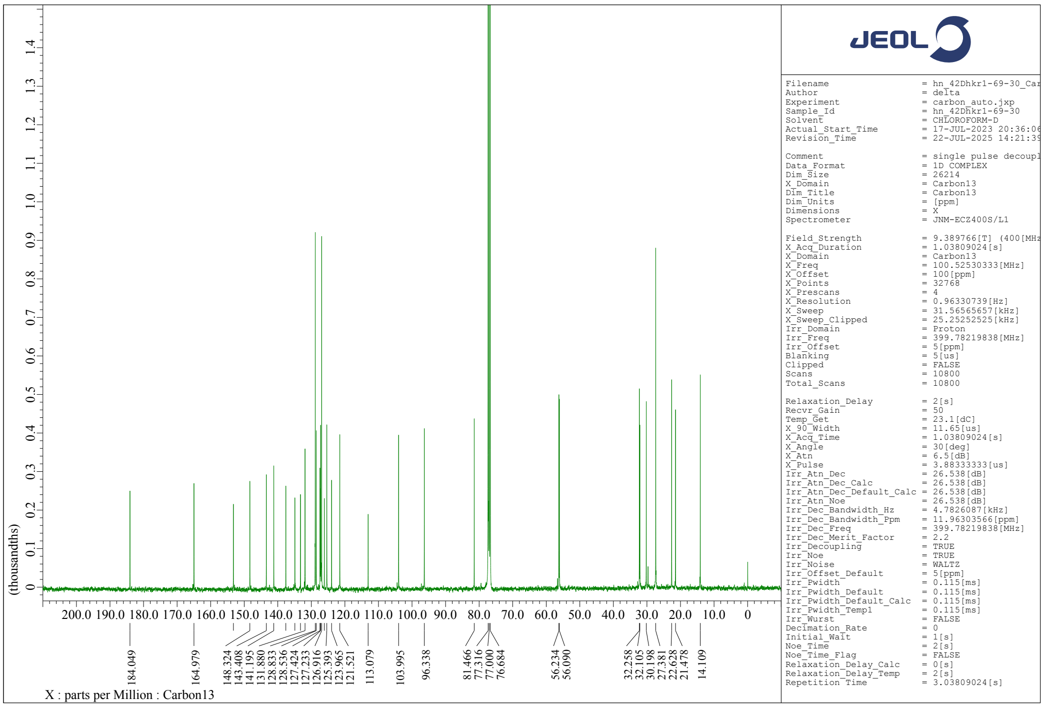

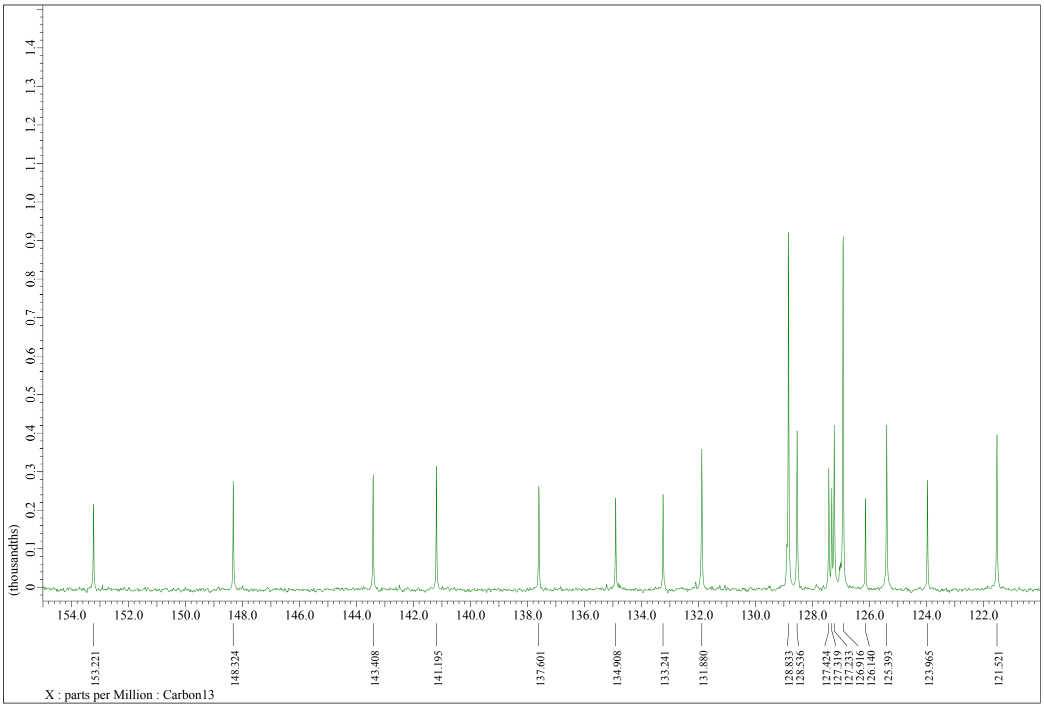

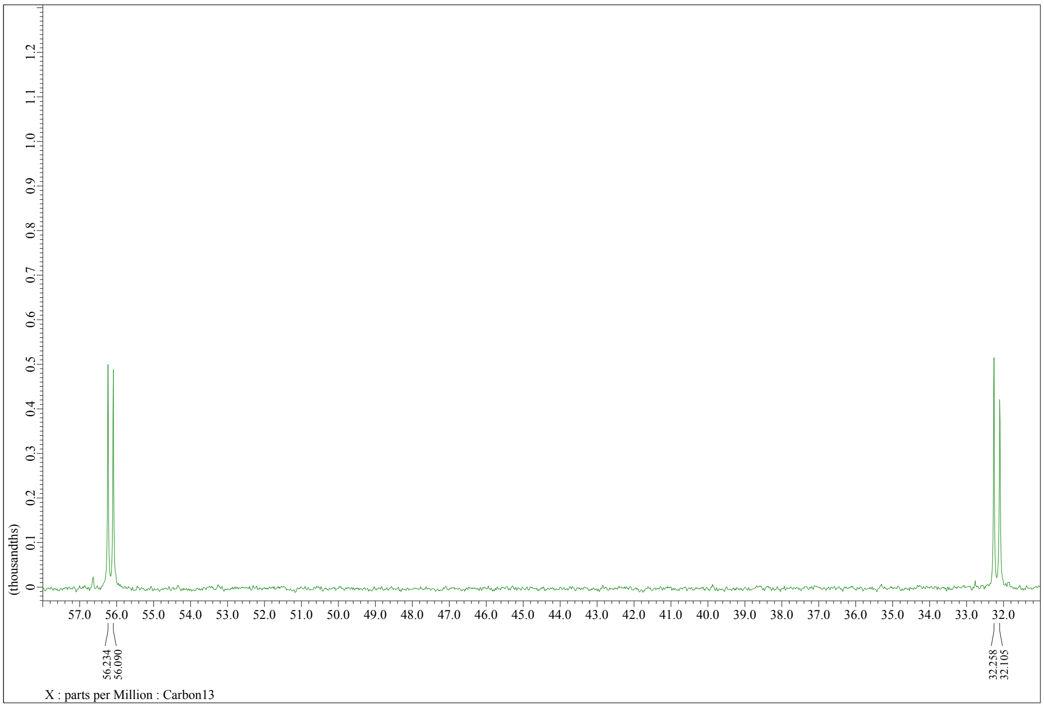

Figure S9. COSY spectrum of **3**

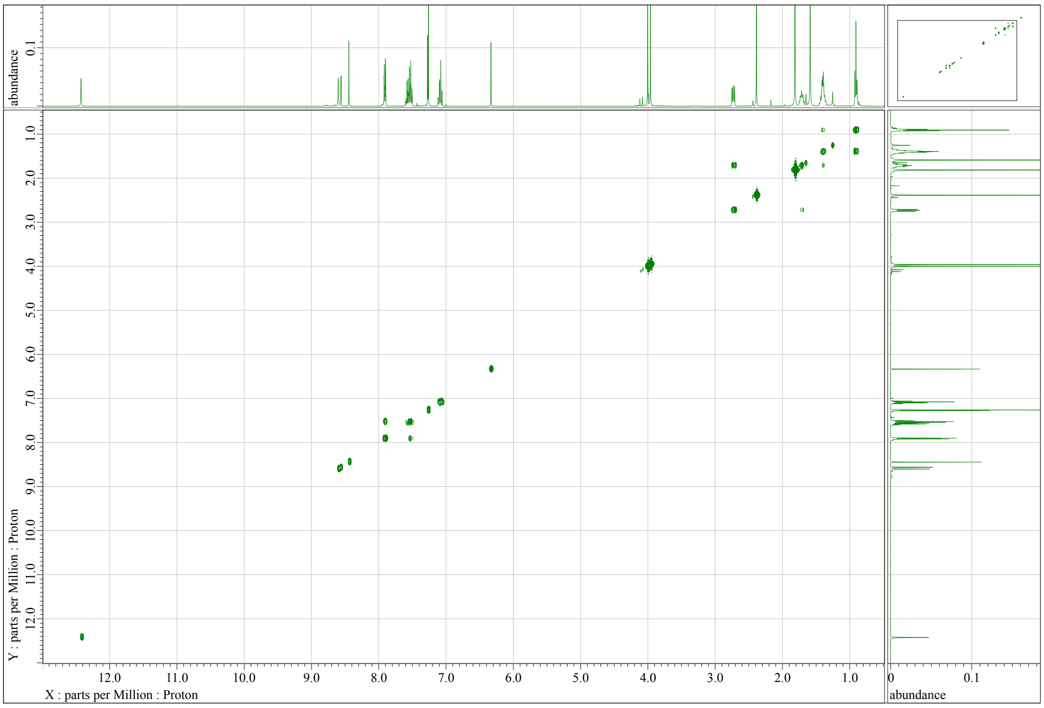

Figure S10. HSQC spectrum of **3**

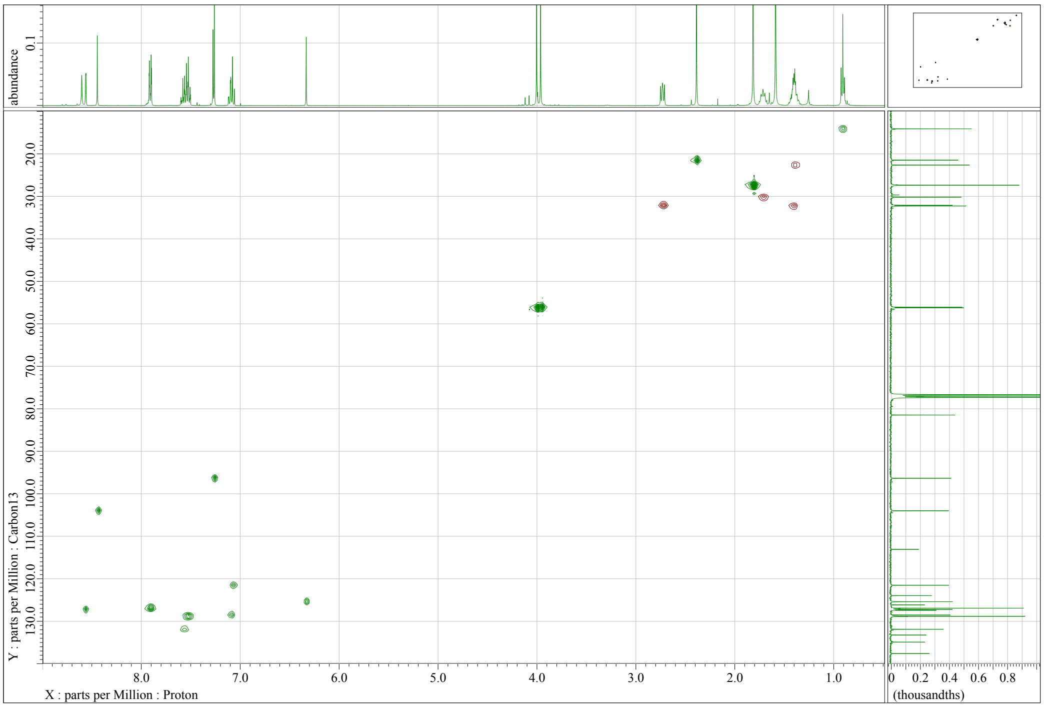

Figure S11. HMBC spectrum of **3**

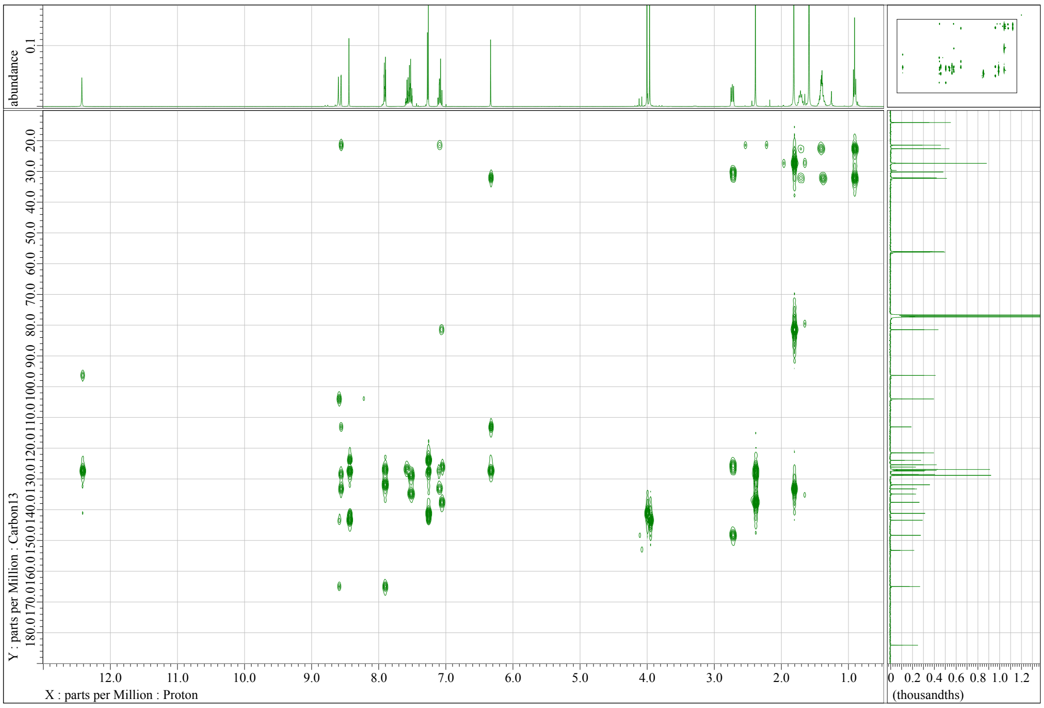

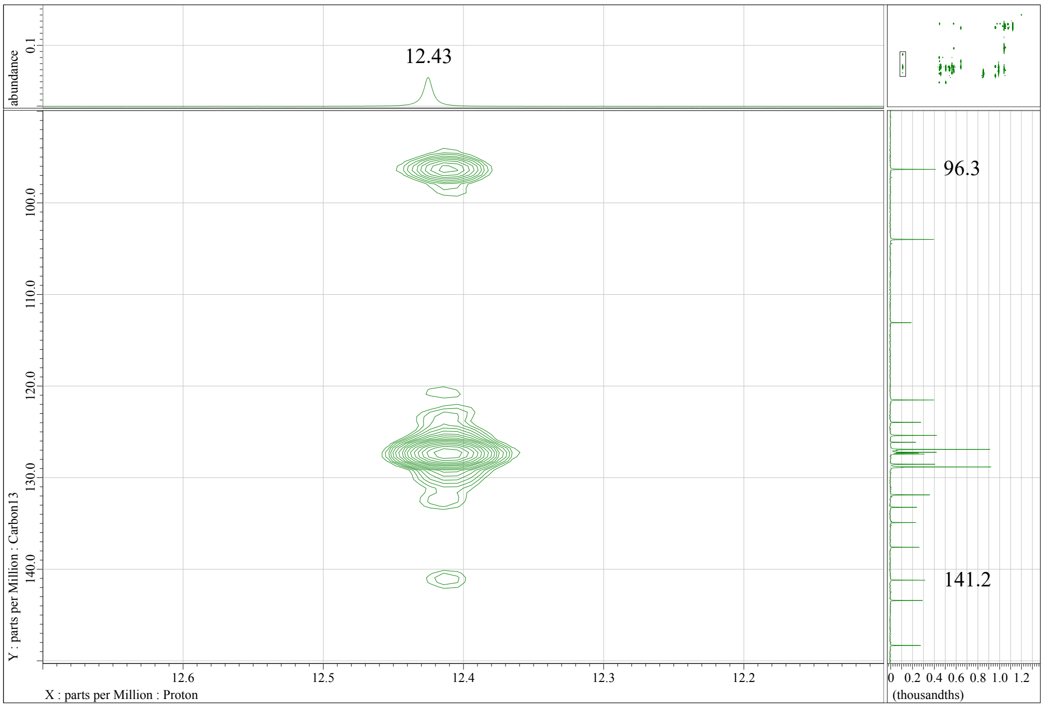

Figure S12. NOESY spectrum of **3**

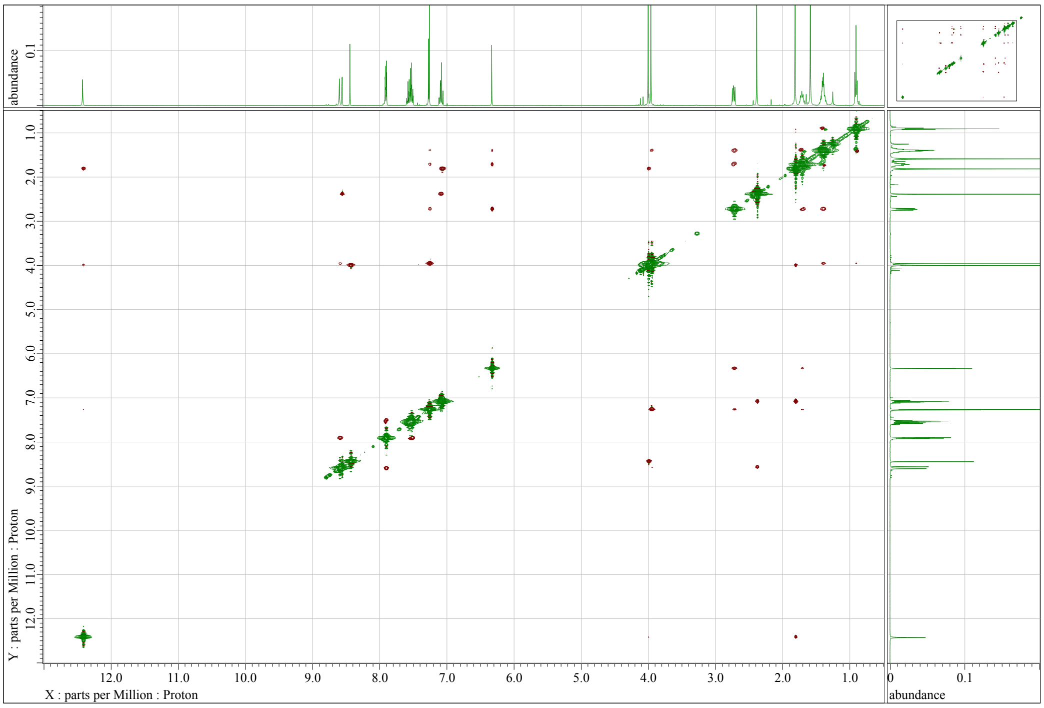

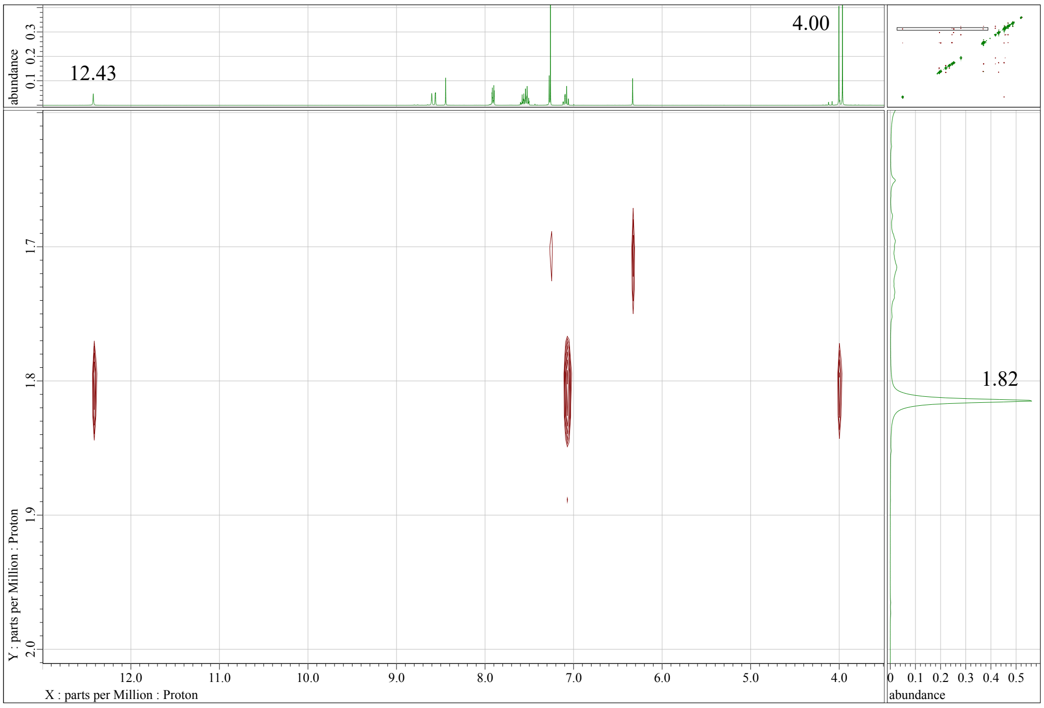

abundance
0.1
0.2
0.3
12.43
4.00
1.82
Y : parts per Million : Proton
1.7
1.8
1.9
2.0
12.0
11.0
10.0
9.0
8.0
7.0
6.0
5.0
4.0
X : parts per Million : Proton
0
0.1
0.2
0.3
0.4
0.5
abundance

3. NMR Spectra of **4**

Figure S13. ^{1}H NMR (400 MHz, $CDCl_3$, ppm) of **4**

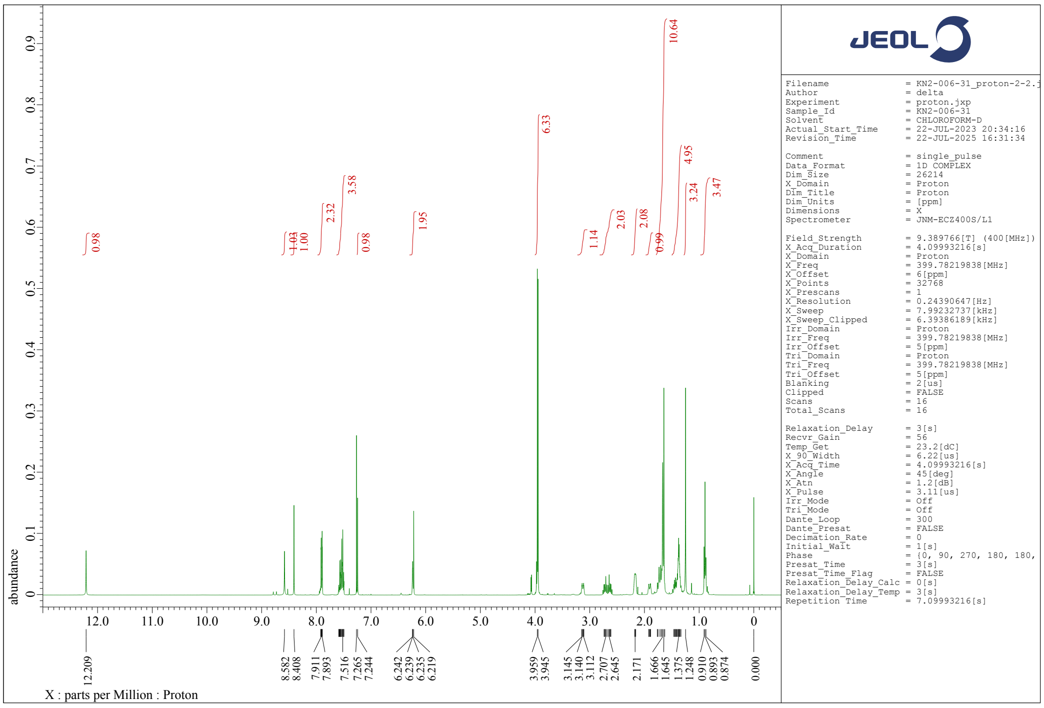

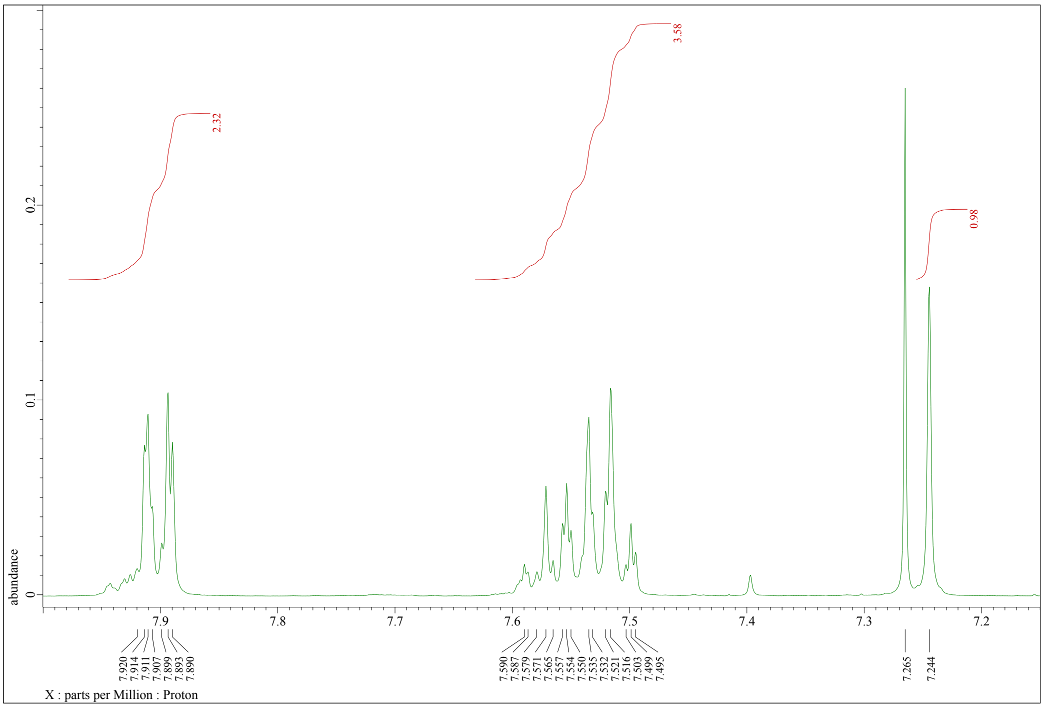

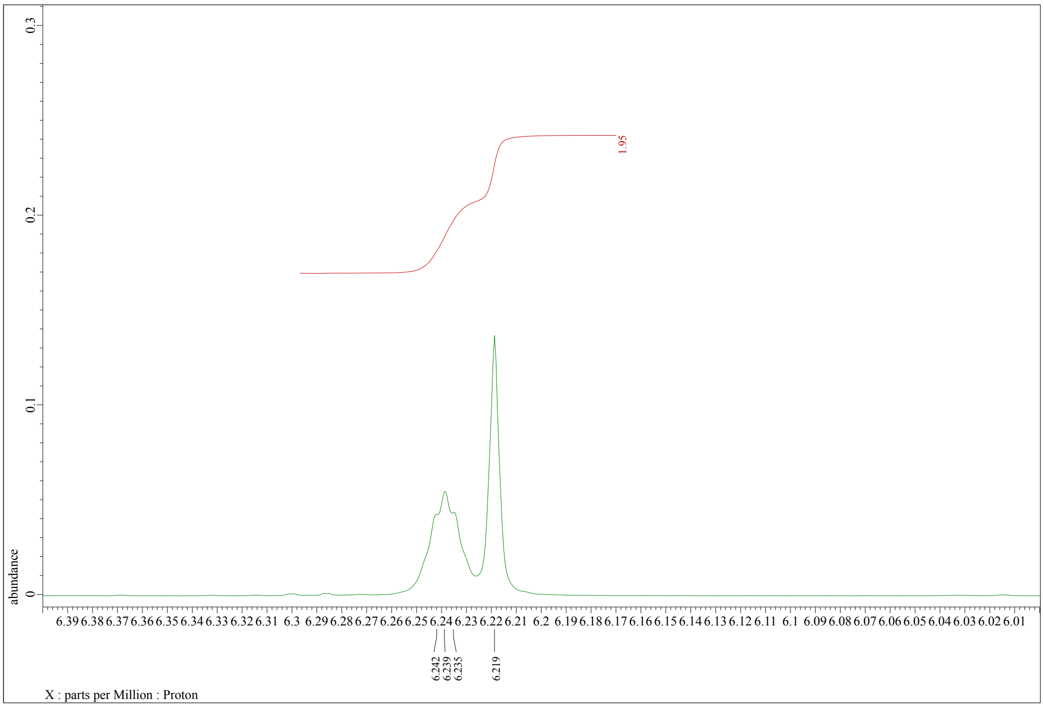

1.95
6.242
6.239
6.235
6.219
abundance
X : parts per Million : Proton

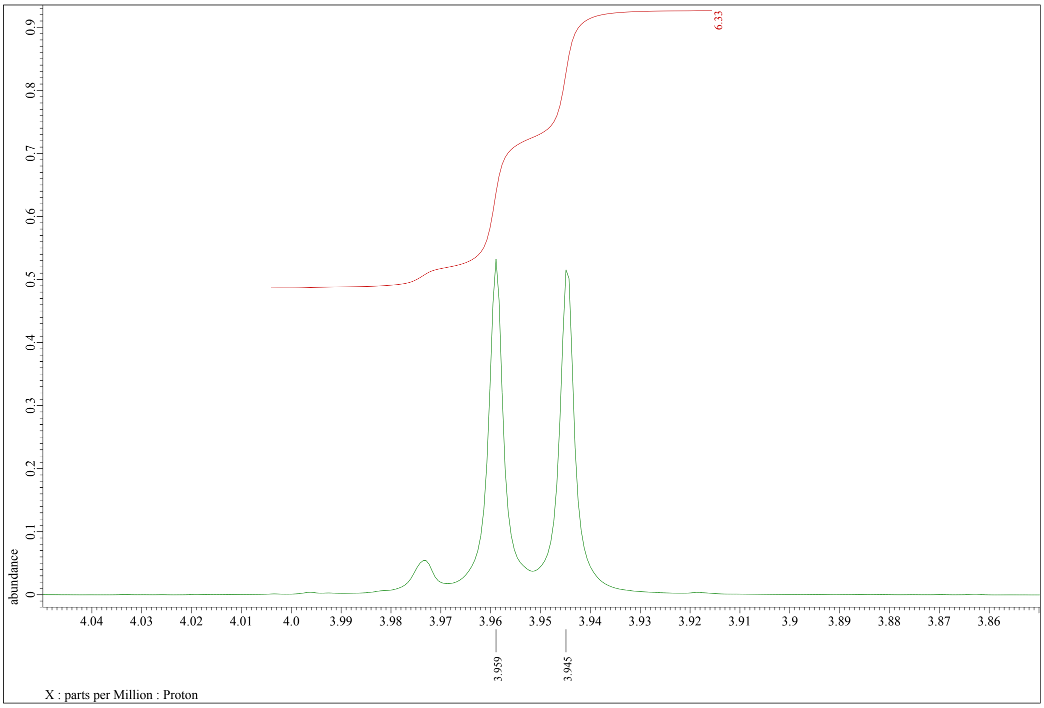

6.33
3.959
3.945
abundance
X : parts per Million : Proton

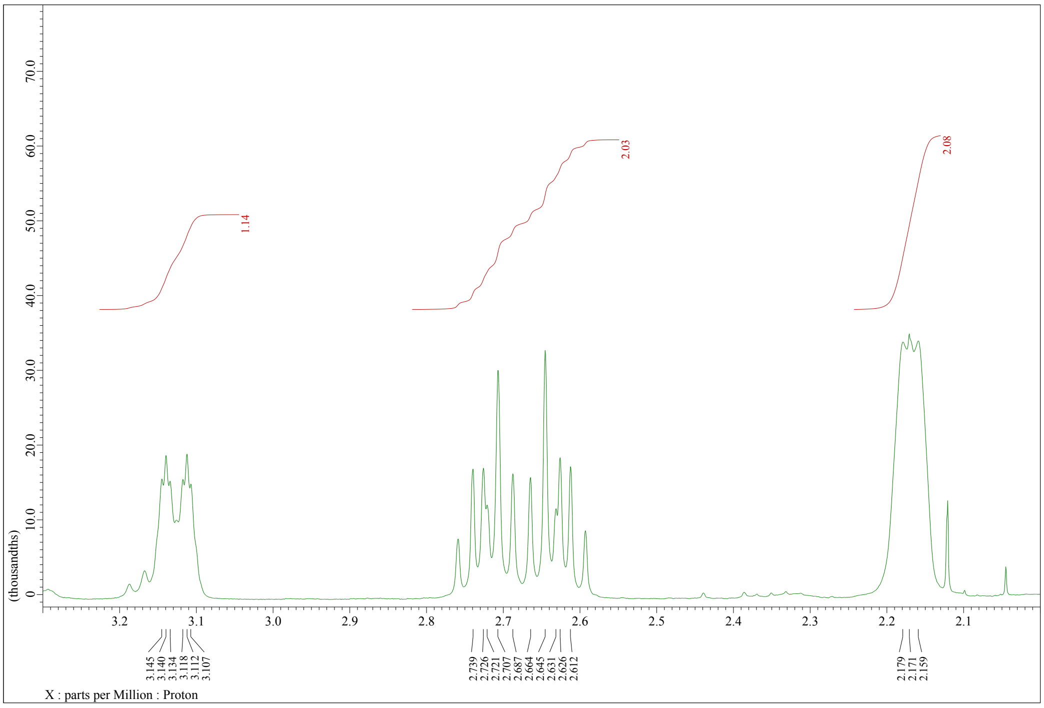

X : parts per Million : Proton
(thousandths)
1.14
2.03
2.08

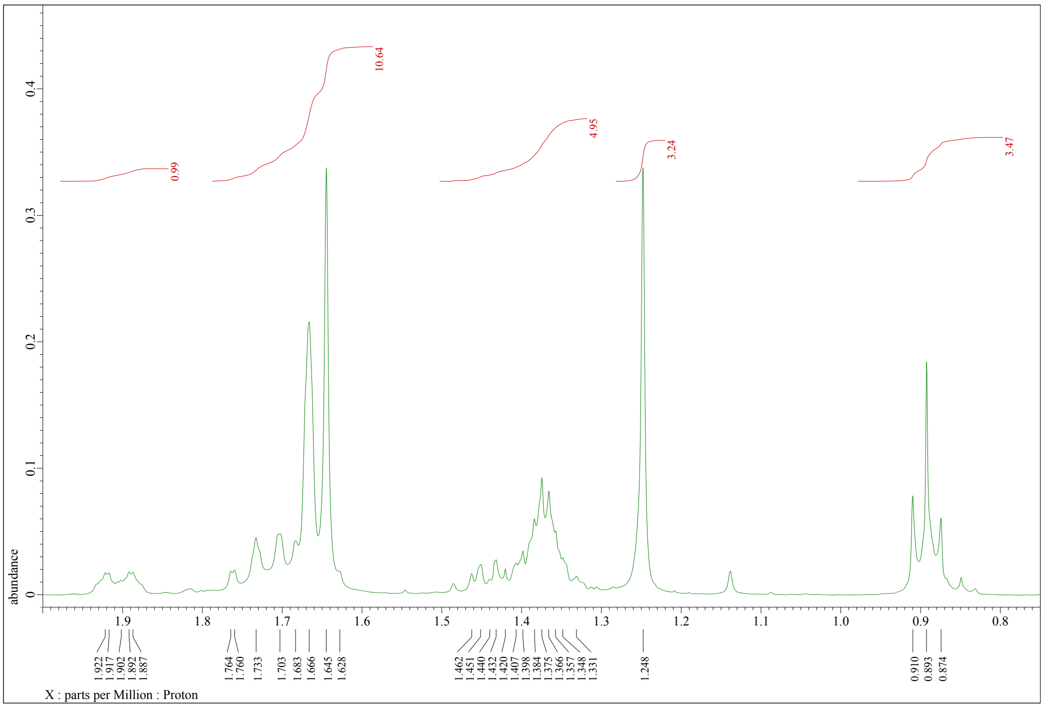

X : parts per Million : Proton
abundance
0.99
10.64
4.95
3.24
3.47

Figure S14. ^{13}C NMR (100 MHz, $CDCl_3$, ppm) of **4**

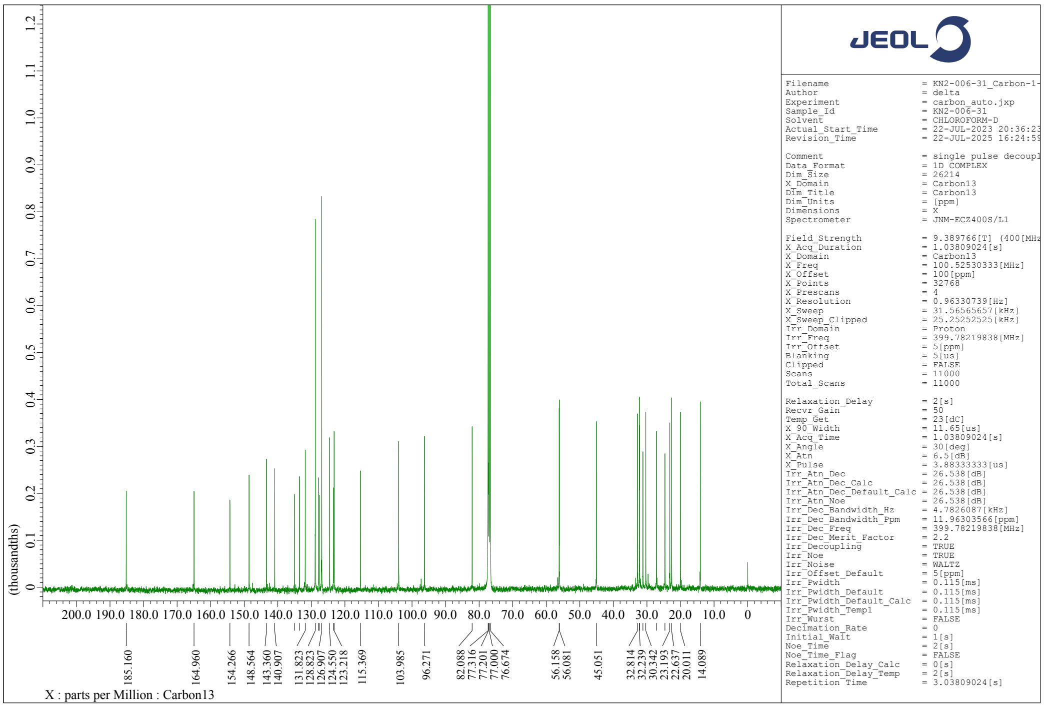

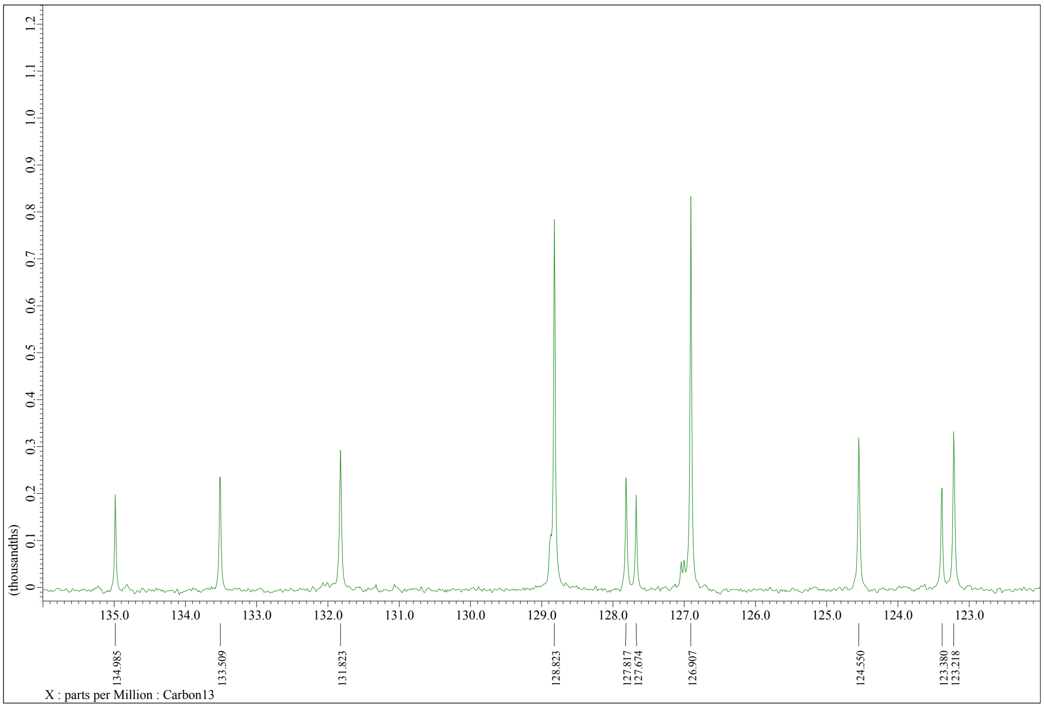

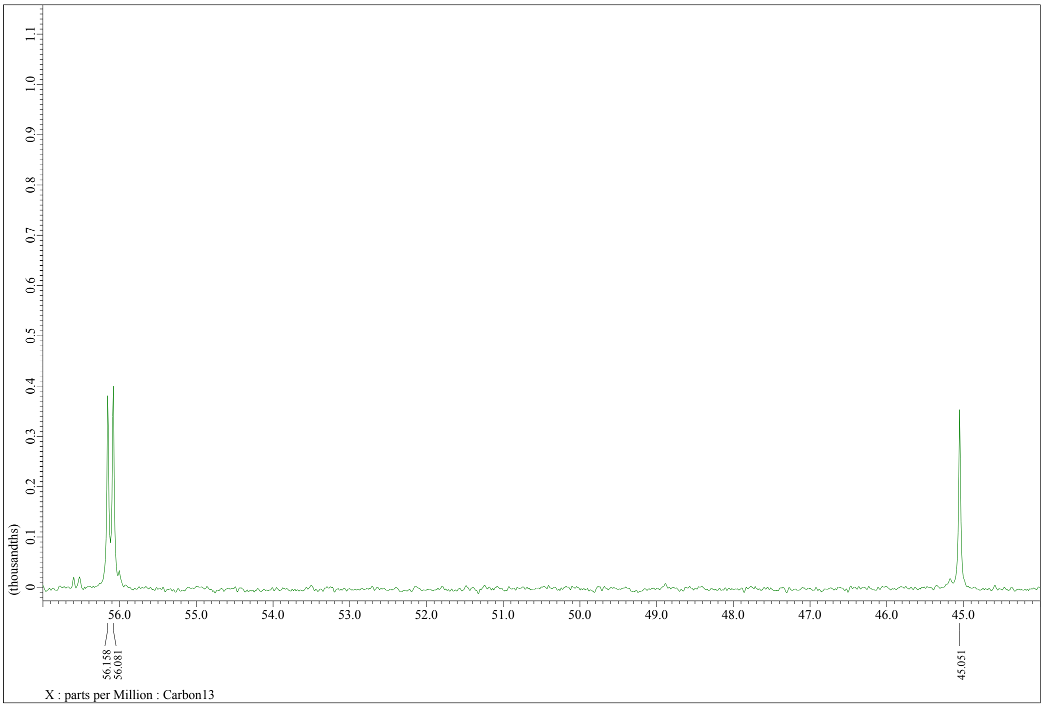

(thousandths)
56.158
56.081
45.051
X : parts per Million : Carbon13

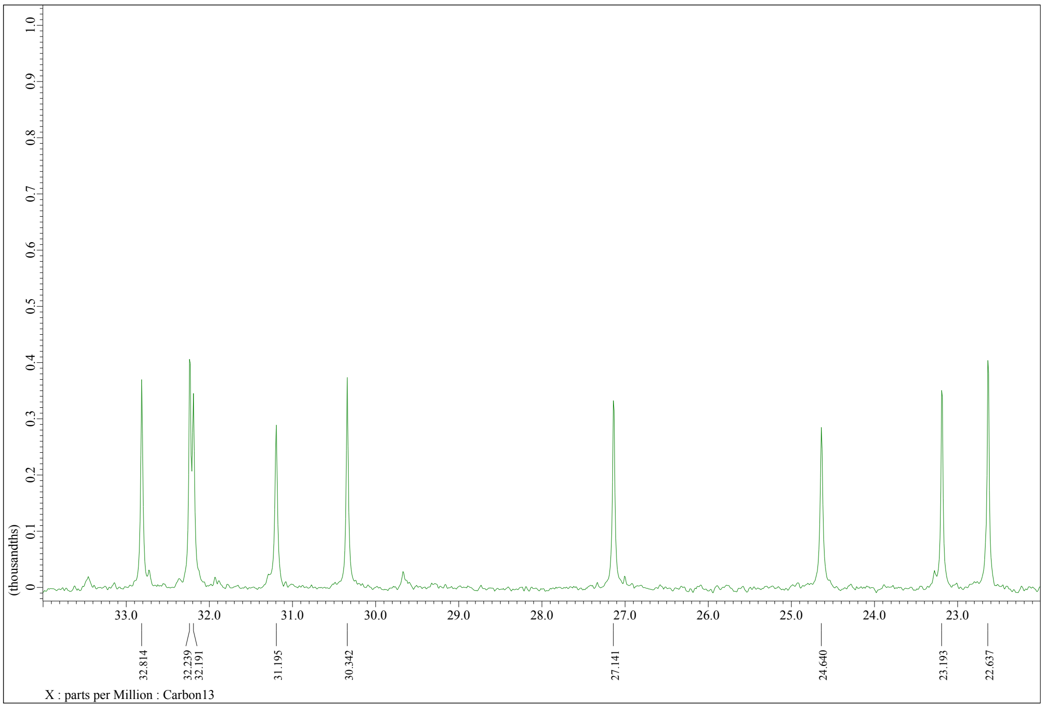

(thousandths)
32.814
32.239
32.191
31.195
30.342
27.141
24.640
23.193
22.637
X : parts per Million : Carbon13

Figure S15. COSY spectrum of **4**

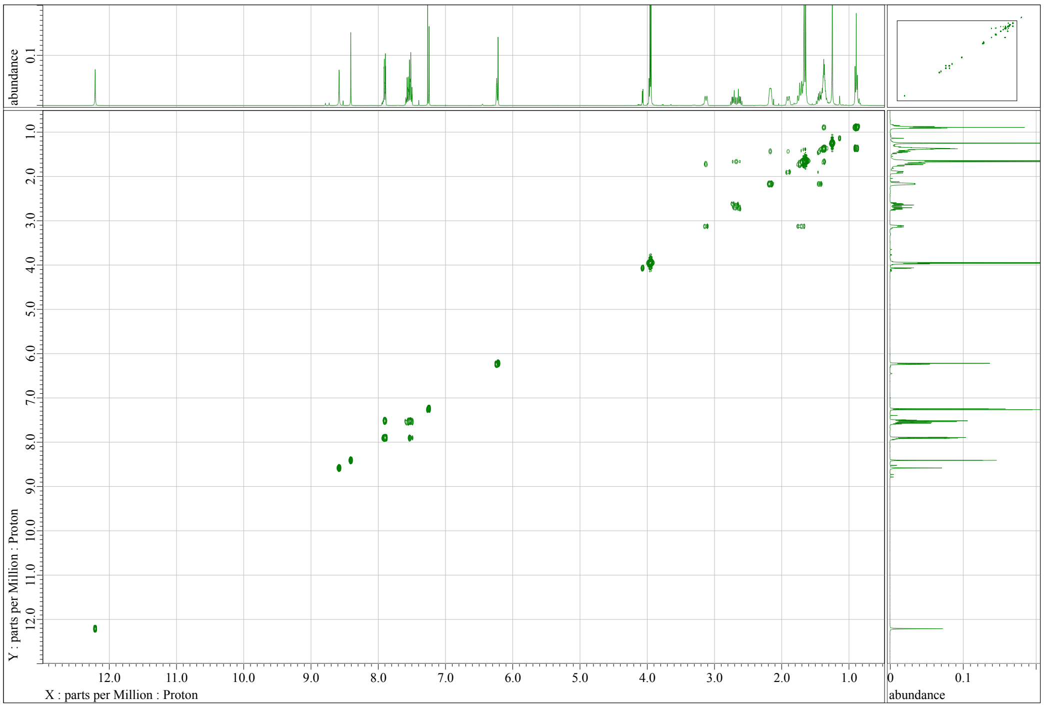

Figure S16. HSQC spectrum of **4**

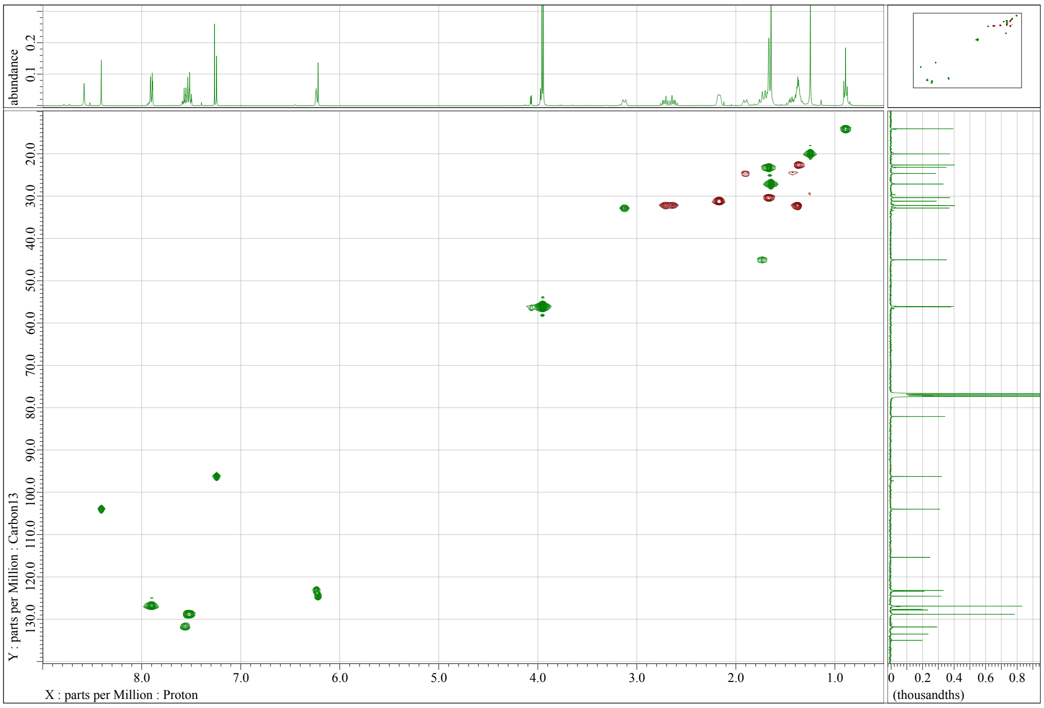

Figure S17. HMBC spectrum of **4**

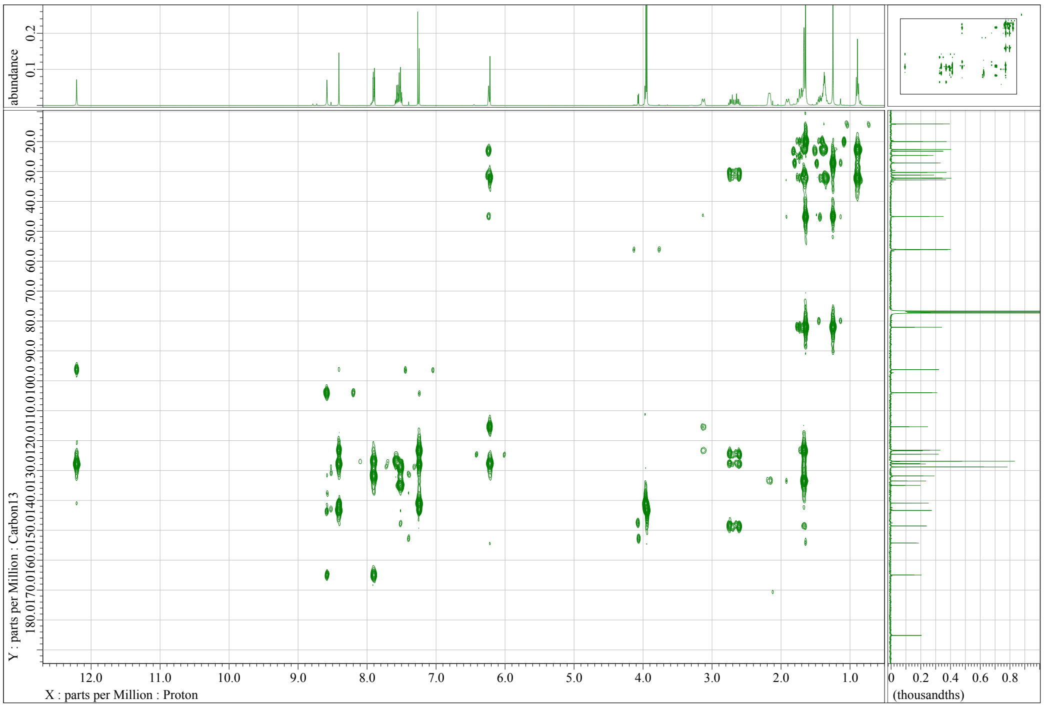

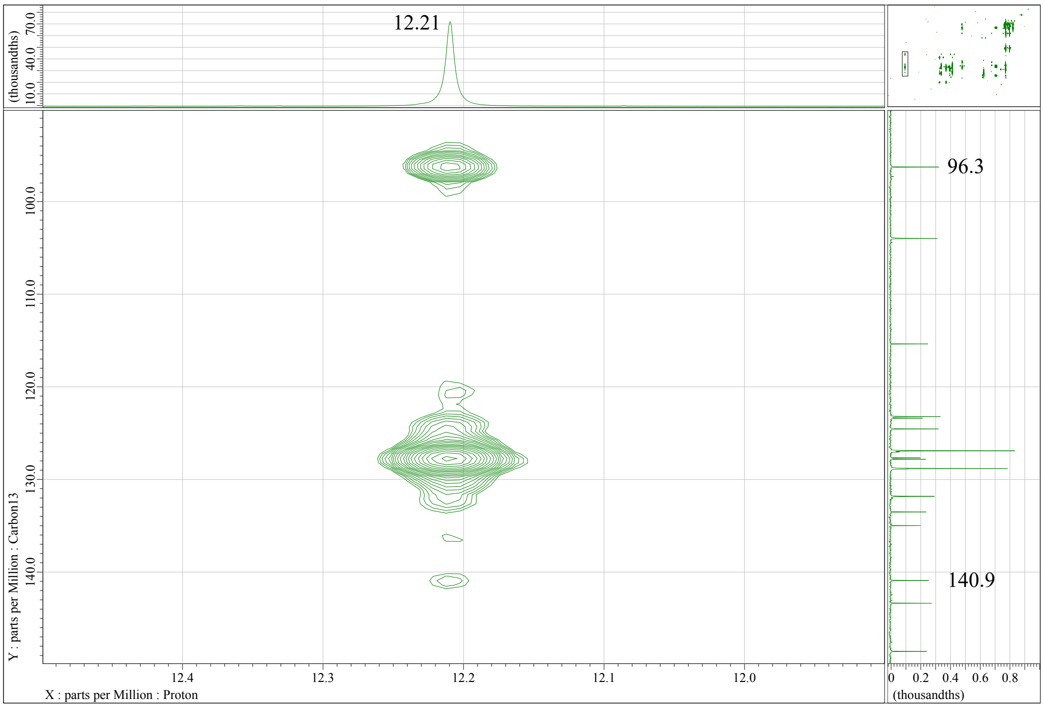

Figure S18. NOESY spectrum of **4**

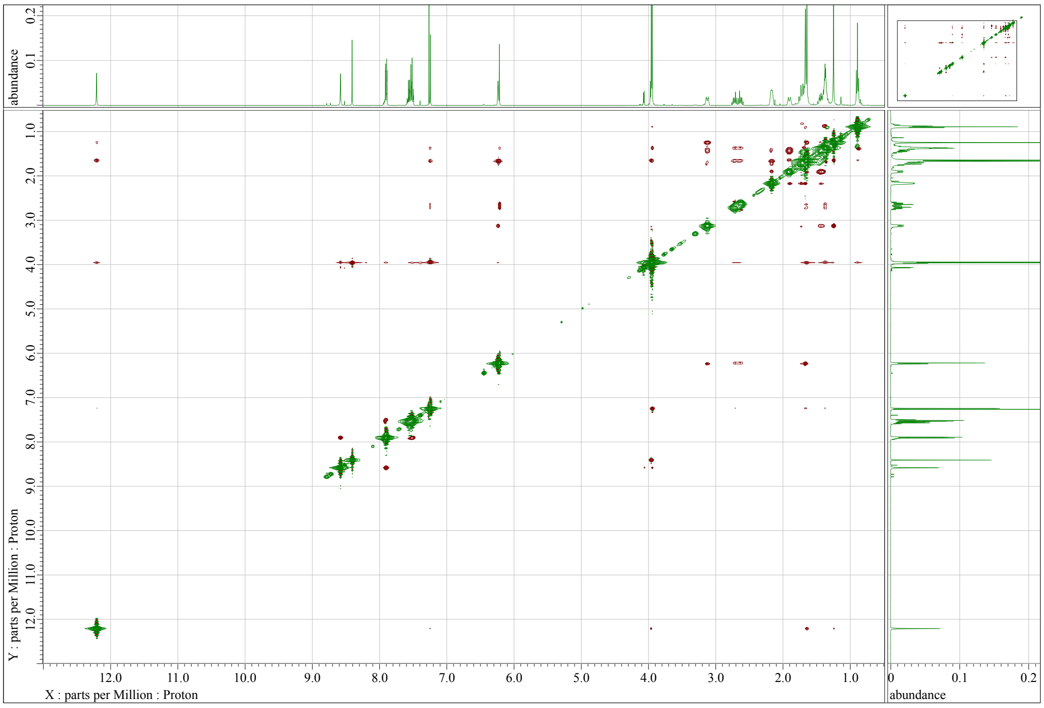

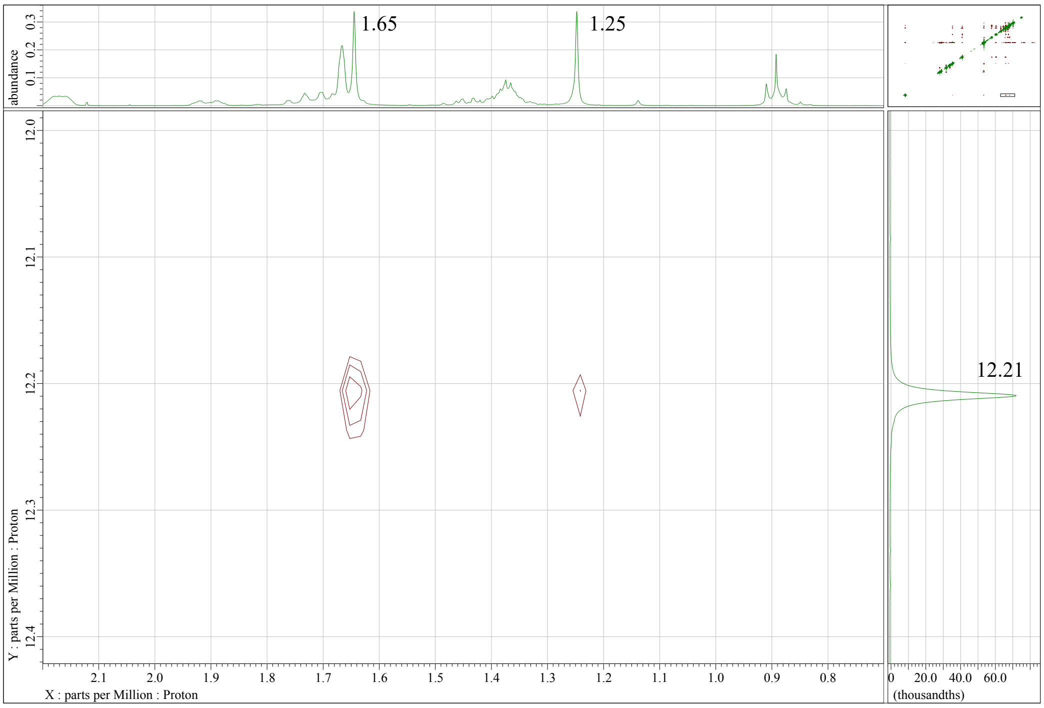

Supplement: Supplementary file 1 [file molecules-30-03462-s001.zip › molecules-3810853-supplementary.pdf]
